# Distribution of Tick-Borne Pathogens in Domestic Animals and Their Ticks in the Countries of the Mediterranean Basin between 2000 and 2021: A Systematic Review

**DOI:** 10.3390/microorganisms10061236

**Published:** 2022-06-16

**Authors:** Baptiste Defaye, Sara Moutailler, Vanina Pasqualini, Yann Quilichini

**Affiliations:** 1 UMR CNRS SPE 6134, Université de Corse Pascal Paoli, F-20250 Corte, France; pasqualini_v@univ-corse.fr; 2 ANSES, INRAE, Ecole Nationale Vétérinaire d’Alfort, UMR BIPAR, Laboratoire de Santé Animale, F-94700 Maisons-Alfort, France; sara.moutailler@anses.fr

**Keywords:** TBPs, pathogens, ticks, domestic animals, Mediterranean Basin, islands

## Abstract

Tick-borne pathogens (TBPs) include a wide range of bacteria, parasites and viruses that cause a large spectrum of animal, human and zoonotic tick-borne diseases (TBDs). The object of this review was to establish an inventory and an analysis of TBPs found in domestic animals in the countries of the Mediterranean Basin. This geographic area occupies a central position between several continents and is an area of movement for animals, humans and pathogens of interest and their vectors, which is important in terms of animal and human health. In this systematic review, we included a total of 271 publications produced between 2000–2021 concerning TBPs in domestic animals. Among this literature, we found a total of 90 pathogen species (known as TBPs) reported in the 20 countries of the area; these were detected in tick species from domestic animals and were also directly detected in domestic animals. In all, 31 tick species were recorded and 12 domestic animal species, the latter comprising nine livestock and three pet species. More than 50% of the publications were from Western Europe. Island data were extracted and assessed, as islands of the Mediterranean Basin were represented in 16% of the publications and 77.8% of the TBPs reported. Our results show the importance of islands in the monitoring of TBPs, despite the low percentage of publications.

## 1. Introduction

Pathogens are one of the largest threats to health worldwide. They can be divided into four main groups: bacteria, parasites, viruses and fungi. These groups are present on all the continents and in the oceans, and target all types of plants and animals as well as humans, depending on their tropism and life cycle [1]. These pathogens have different transmission strategies: (i) direct contact between individuals from the same species or between individuals from different species; (ii) indirect contact through the environment or dissemination in the air; and (iii) through vectors such as hematophagous arthropods, such as mosquitoes, ticks or sandflies that can transmits pathogens via blood-sucking from one host to another [2,3].

Among the different pathogens, many are zoonotic and are transmitted between animals and humans, while others are non-zoonotic and specifically target either humans or animals. Zoonotic pathogens account for about 60% of pathogens worldwide and are particularly present in emerging diseases [4]. Transmission of these pathogens by hematophagous insects has become one of the main threats to global health in recent decades [4].

Hematophagous arthropod vectors can transmit pathogens called vector-borne pathogens (VBPs), which may be zoonotic or non-zoonotic, from one individual to another. They can transmit bacteria, parasites and viruses [5]. For example, the role of ticks in the circulation of Lyme disease between animal and human populations in North America has been established [6]. Annually, vector-borne diseases (VBDs) cause more than 17% of infections and over 700,000 deaths across the globe [7]. The two predominant vectors, in order of importance, are mosquitoes and ticks. However, ticks also contain bacterial endosymbionts, such as *Rickettsia*-like, *Francisella*-like or *Coxiella*-like organisms. These are endosymbiotic intracellular bacteria that are harmless to mammals and may be required for tick survival [8]. In previous research, they may have been misidentified as pathogens harmful to human and animals. 

Ticks (Ixodida) rank first for veterinary vector-borne pathogens and second for human pathogens, just after mosquitoes [1,9]. Ticks are hematophagous arthropods of the *Ixodida* order. They are composed of three families distributed across the world. The *Ixodidae* family is divided into the Prostriasta (subfamily Ixodinae, genus *Ixodes*) and Metastriata (all other subfamilies, genera: *Anamalohimalaya*, *Cosmiomma*, *Dermacentor*, *Hyalomma*, *Margaropus*, *Nosomma*, *Rhipicentor* and *Rhipicephalus*). These are also named “hard ticks”, and the family includes most species. The *Argasidae* family is divided into two subfamilies, the Argasinae (genus *Argas*) and the Ornithodorinae (genera: *Ornithodoros*, *Otobius* and *Carios*), also named “soft ticks”. The *Nuttallielliedae* family is composed of just one species [10]. The tick’s life cycle is divided into four stages: egg, six-legged larva, eight-legged nymph without sexual organs, and eight-legged adult male or female. Tick tropism depends on the species and stage. They can be monotropic, ditropic or teleotropic, with one, two or several hosts, respectively. They can transmit pathogens by feeding on a broad spectrum of terrestrial species, including wild animals (mammalian, avifauna, reptilian and amphibian), livestock and pets. These pathogens are named tick-borne pathogens (TBPs) and are known to be both pathogens of veterinary importance and zoonotic pathogens harmful for humans [1]. 

Ticks can transmit a wide range of bacteria, parasites and viruses [1]. An example is Lyme disease in Europe. This zoonotic disease is caused by spirochetes bacteria from the group *Borrelia burgdorferi sensu lato* and the genus *Borrelia*, and could be transmitted by a wide range of ticks [11]. In Europe, this includes *Borrelia burgdorferi sensu stricto*, *Borrelia afzelii* and *Borrelia garinii*, with approximately 65,500 cases every year [6]. One reported tick-borne virus is the Crimean-Congo Hemorrhagic Fever virus from the genus *Orthonairovirus*, which is responsible for many outbreaks internationally, with a high fatality rate of 40% [12]. It is mainly transmitted by ticks of the genus *Hyalomma*, which act as both vector and reservoir [13]. Regarding parasites, causative pathogens such as the *Babesia* genus are transmitted by ticks and can threaten human and veterinary health [14]. 

Given its geographic position between Europe and Africa, the Mediterranean Sea is bordered by a significant number of countries with a high variation of biotopes, ranging from Mediterranean to arid climates. The Mediterranean Basin is an area highly affected by climatic change, animal migration and human activity [15,16]. While increasing temperatures in Northern Europe favor the prevalence of ticks such as *Ixodes ricinus*, in the Mediterranean Basin the development of dry areas with arid types of vegetation favors the proliferation of ticks such as *Hyalomma marginatum* [17]. This development is also supported by the feeding of immature-stage ticks on birds and the long duration of their attachment to hosts during these stages [18]. This supports the theory of the key role of migratory birds in tick dissemination [19]. Other migration, such as the movement of dogs without restriction in the Mediterranean Basin, also favors the circulation of *Rhipicephalus sanguineus* s.l. [20].

We focused our review on the countries of the Mediterranean Basin where the different TBPs detected in domestic animals and their ticks were screened. Moreover, in order to compare the geographical distribution of these TBPs, and to highlight possible changes in their spread in the future, four areas were considered: Western Europe, composed of France, Italy, Malta, Monaco and Spain; the Balkans, composed of Albania, Bosnia-Herzegovina, Croatia, Greece, Montenegro and Slovenia; the Middle East, composed of Cyprus, Israel, Lebanon, Palestine, Syria, and Turkey; and finally North Africa, composed of Algeria, Egypt, Libya, Morocco and Tunisia. The different areas were determined by common biotope and geographic proximity. The last part of our review was to investigate the potential role of the western and eastern islands in the monitoring of TBPs in domestic animals and their ticks, according to their geographic position, surface areas and potential role in TBP circulation through animal migration. In this part, we focused on the distribution of TBPs in the Mediterranean islands in order to determine a potential role of the islands in the distribution of TBPs.

The aim of this study was to review, according to PRISMA guidelines, the literature published between 2000 and early 2021 addressing the presence of TBPs on domestic animals and their ticks in the countries of the Mediterranean Basin, with the following objectives: 

Perform a bibliometric analysis of TBP studies.

Review the diversity of TBPs, positive engorged tick species, domestic animal hosts of TBPs and positive tick species. 

Compare the distribution of TBPs from domestic animals and their ticks in the four main areas defined.

Focus on the distribution of TBPs in the Mediterranean islands.

## 2. Materials and Methods

We undertook a literature review concerning tick-borne pathogens in all countries of the Mediterranean Basin (*n* = 20). We followed PRISMA guidelines and used explicit and systematic methods to identify, select and evaluate the studies relevant to the topic [20]. We compiled and evaluated the data from the studies included in this review. 

**Figure 1:**
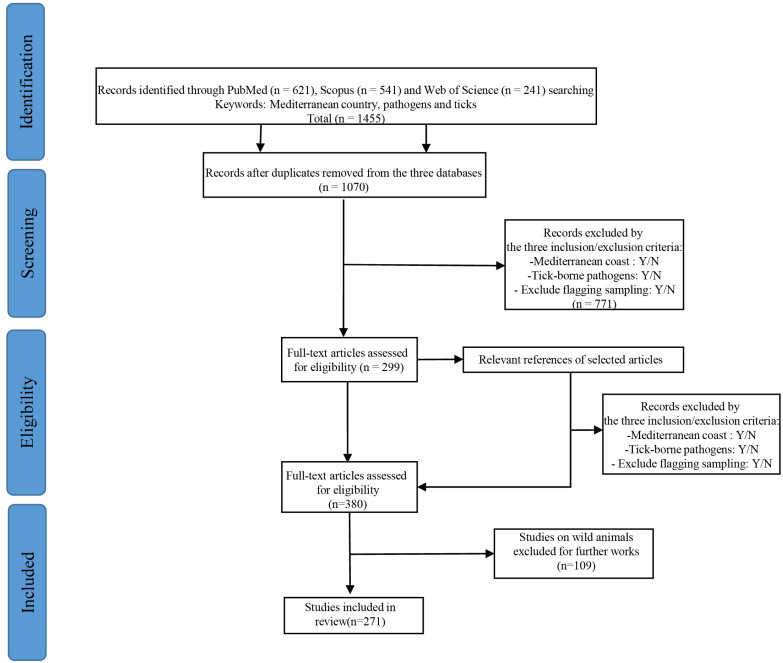
Methodological diagram of the bibliographic research following the PRISMA 2009 Flow according to Moher et al., 2015 [21].

All articles published in English in international journals indexed by PubMed, Scopus and Web of Science were considered (Figure 1). The date range was from 1 January 2000 through 31 February 2021. We used keywords for each country of the Mediterranean Basin, classified in alphabetical order: Albania OR Algeria OR Bosnia-Herzegovina OR Cyprus OR Croatia OR Egypt OR France OR Greece OR Israel OR Italy OR Libya OR Malta OR Monaco OR Montenegro OR Morocco OR Palestine OR Slovenia OR Spain OR Tunisia OR Turkey AND Pathogens AND Ticks, with the option “all fields” to recover articles in which search items appeared in the title, abstract and keywords. First, all papers considered to be “grey” literature, such as literature reviews, case studies, manuscripts and abstracts of posters from conferences and guides from relevant organizations, were excluded. We also discarded clinical descriptions of disease and diagnosis in humans and animals. We selected all papers focusing on the distribution and circulation of tick-borne pathogens and their vectors. We focused on studies dealing with the distribution of these pathogens and ticks in the countries of the Mediterranean Basin and their islands. Second, we excluded duplicate and inaccessible articles (due to language or unavailable full text). We reviewed the titles and abstracts and applied inclusion/exclusion criteria on 1070 articles. Filtering was carried out by responding to the following questions:

1. Did the study include a country with a Mediterranean coast: Yes/No
2. Did the study include tick-borne pathogens: Yes/No
3. Did the study exclude ticks collected in vegetation: Yes/No

Only publications considering TBPs in domestic animals and/or TBPs in engorged ticks collected on domestic animals in Mediterranean countries were included. This made it possible to conduct an overview of research about TBPs potentially infesting and circulating in the domestic animal population only. The articles were saved if the answers to the three questions were “yes”; otherwise they were eliminated. For the next step, we reviewed the full-text and entered the information of interest into a database for 299 articles. We also excluded articles that we found did not fit the criteria after reading the full text.

We reviewed the bibliography of each selected article in order to check for new articles to include in the review and relevant articles in the field of research. We followed the same steps as previously described for new articles (*n* = 81). 

For the last step, we excluded articles dealing with wild animals (*n* = 109) and retained only articles dealing with domestic animals (*n* = 271). The selection steps are summarized in Figure 1, with an explanation of the inclusion/exclusion of articles for this review.

The data of interest were captured in an Excel table that was tested in advance with 15 articles, and included the following information:

1. Main characteristics of the studies: article ID, years, authors, analytical and statistical methodology
2. Pathogen-related information: pathogens screened and detected, species, number of species, zoonotic status, host
3. Tick-related information: species, type, number, stage
4. Host-related information: groups, sedentary or migratory
5. Area of interest: country, type of area, number of sampling sites

The different outputs of the data worksheet were selected following mutual agreement from all the authors.

## 3. Results

### 3.1. Bibliographic Analysis

**Figure 2:**
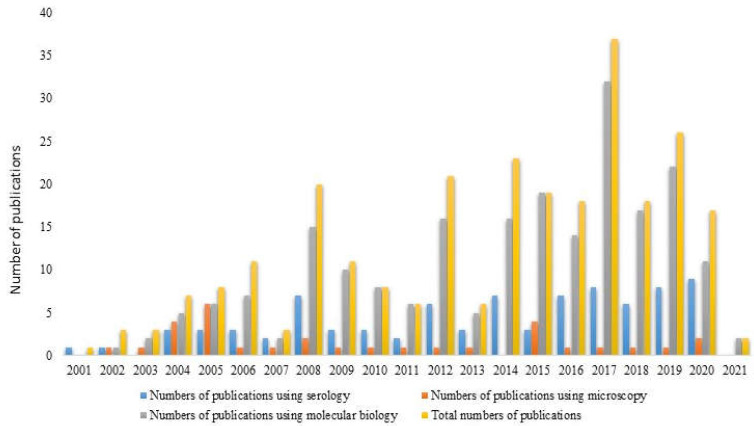
Number of publications through the years (2000–February 2021). The total number is indicated in yellow, and the other colors indicate the number of publications according to the detection method used (serology; microscopy; molecular biology).

The distribution of the publications through the years is shown in Figure 2. During the first decade, the number of papers increased slightly until 2008, with a maximum of 20 publications, and decreased in the years 2009 and 2010 (11 and nine papers, respectively). During the second decade, there was first a low rate of publication, except in 2012, with a peak of 21 publications. Between 2014 and 2021, the mean number of published papers per year was approximatively 20, accounting for 60% of the published papers about tick-borne pathogens on domestic animals, with a peak of 37 papers in 2017. In 2021, the number of papers was low because we stopped the research in February 2021. The rate of publication of research on tick-borne pathogens increased notably during this period.

This trend can be explained by different factors:

First, there is increased interest from the scientific community due to the importance of this subject in terms of human and animal health.

Second, there is improved accessibility of scientific journals and publications.

Third, there have been changes in pathogen detection methodology through the years, and particularly developments in the field of molecular biology. 

Concerning this last point, tick-borne pathogens were typically detected using three kinds of methods (Figure 2). The first category of method was serological analysis, involving detection of pathogens in blood and tissue samples by way of antibodies; such methods include ELISA and immunofluorescence. The second category was the microscopy approach, which is more common for parasites. The third category was molecular biology, which covered techniques involving the detection of pathogen nucleic acids in both tick and animal hosts, such as polymerase chain reaction (PCR), next-generation sequencing (NGS), and high-throughput sequencing techniques. Even though researchers may use more than one approach to detect and monitor TBPs, we observed that over the years, molecular biology rapidly became the main analytical approach. The increase in publications over the last ten years seems to be linked to the development and accessibility of molecular biology techniques, and to their ability to simultaneously detect a large number of pathogens. 

**Figure 3:**
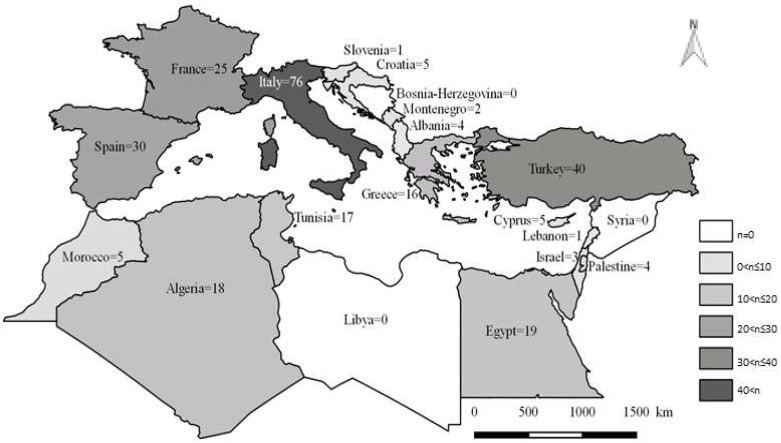
Number of publications dealing with TBPs in domestic animals and their ticks in the Mediterranean Basin according to country of origin.

In addition to changes in the amount of data available depending on the year, we can also note variability of these data according to their geographic origin. In total, 271 publications and 20 countries of the Mediterranean Basin were considered in this review. The mean number of publications per country was about 13, and the number of publications varied from 0 to 76, depending on the country (Figure 3). 

### 3.2. Tick-Borne Pathogens in Countries of the Mediterranean Basin

A total of 271 publications were analyzed: 56.5% were about bacteria, 37.7% were about parasites and 5.80% were about viruses. A total of 90 pathogens from 18 genera were detected in domestic animals and their ticks from the Mediterranean Basin: 11 genera of bacteria, four genera of parasites, and three genera of viruses (Table 1). Among these 90 TBPs, 60% were zoonotic and 40% were non-zoonotic or of unknown status (Table 1). Among the 90 TBPs detected, 73.3% were found in ticks and 76.7% were found in animal hosts. Of the TBPs reported, 50% were detected in both (51.1%).

#### 3.2.1. Parasites

##### Nematoda 

*Cercopithifilaria*

Pathogens in the genus *Cercopithifilaria* are microfilariae parasites that mainly infect wild and domestic animals. Among the three species *Cercopithifilaria grassii*, *Cercopithifilaria* spp. *sensu* and *Cercopithifilaria bainae*, only the latter was reported in our research [236]. It was detected in two countries: Greece, with a prevalence of 7% from *Rh. sanguineus* s.l. collected from dogs; and Italy, at 25.86% from dogs [22,23]. This parasite was found in both ticks and animals, but from two distinct publications. The genus was found in only 0.7% of the publications and 1.9% of the publications concerning parasites. 

##### *Apicomplexa* 

*Babesia*

*Babesia* is a genus of erythrocytic protozoal parasites transmitted by ticks that cause babesiosis in both animals and humans. The main *Babesia* species transmitted by ticks are *B. divergens*, *B. duncani*, *B. microti*, *B. venatorum. B. vogeli* and *B. canis* (responsible for canine babesiosis). *Babesia vogeli* was the species found in the largest range of countries among the 15 *Babesia* species found in domestic animals. *Babesia vogeli* was found in seven countries (Croatia, Cyprus, France, Italy, Palestine, Spain and Turkey) and was not detected in countries of North Africa. Among these seven countries, the highest prevalence of *B. vogeli* in animals was 14% from dogs in Italy and 10.5% in *Rh. sanguineus* s.l. ticks collected from dogs in France [56,59]. The next most commonly found species were *B. canis* and *B. ovis*. *Babesia canis* was found in five countries (Croatia, France, Italy, Spain and Turkey). The highest prevalence was 71.4% from dogs in Italy along with 5.65% from *Rh. sanguineus* s.l. and *Dermacentor reticulatus* from dogs in France. *Babesia ovis* was found in five countries (Algeria, Italy, Palestine, Spain and Turkey). The genus *Babesia* is one of the most frequently screened or found pathogens, along with *Rickettsia* and *Anaplasma*, featuring in 17.7% of the publications. It was the most commonly screened or found of the parasites, featuring in 46.2% of the publications concerning parasites. It showed the highest species diversity, just after the genus *Rickettsia*.

*Hepatozoon*

Parasites in the genus *Hepatozoon* are intracellular protozoa belonging to the phylum Apicomplexa that infect amphibians, birds, mammals and reptiles [237]. Two species were found in domestic animals from countries on the Mediterranean Rim. *Hepatozoon canis* was found in cats or dogs in eight countries (Croatia, Cyprus, France, Greece, Italy, Palestine, Spain and Turkey). The highest prevalence was 22.3% in dogs along with 20.58% in *Rh. sanguineus* s.l. from dogs, both occurring in Turkey [80,238]. The second species was *Hepatozoon felis*, with the highest prevalence found to be 5.1% from cats in Italy and 1.7% from *Rh. sanguineus* s.l. from dogs in Turkey [75,80]. The genus *Hepatozoon* was the third most commonly found or screened parasite, featuring in a total of 7% of the overall publications and 18.2% of the publications concerning parasites. Pathogens in the genus *Hepatozoon* were found only in two pet hosts (cats and dogs).

*Theileria*

Along with *Babesia* and *Hepatozoon*, the genus *Theileria* belongs to the phylum Apicomplexa, and with *Babesia*, also to the piroplasmids group. Species in this genus infect mammals and have an obligatory cycle in ticks. They cause benign to fatal theileriosis in breeding animals [239]. A total of 11 species were found in domestic animals from the Mediterranean Basin: *T. annae*, *T. annulata*, *T. buffeli*, *T. cervi*, *T. equi*, *T. lestoquardi*, *T. luwenshuni*, *T. orientalis*, *T. ovis*, *T. uilenberg* and *T. sergenti*. The species found in the most countries were *T. ovis*, *T. annulata* and *T. buffeli*. *T. ovis* was found in six countries (Algeria, France, Greece, Palestine, Spain, and Turkey), as was *T. annulata* (Algeria, Egypt, Italy, Spain, Tunisia and Turkey), while *T. buffeli* was found in five countries (Algeria, France, Italy, Spain, and Turkey). The highest prevalence of *T. ovis* was 53.3% in goats and sheep from Algeria, along with 37.35% in ticks from the genus Rhipicephalus taken from goats and sheep in Algeria [44,66]. *T. annulata* was found in 64% of cattle from Turkey and in 5% of *Rh. (Bo.) annulatus* collected from cattle, goats and sheep from Algeria [44,92]. The highest prevalence of *T. buffeli* was 11.56% from cattle in Turkey along with 2.8% in three tick species, *I. hexagonus*, *I. ricinus* and *Rh. sanguineus* s.l., from cattle in Italy [60,240]. Similarly to *Babesia*, the genus *Theileria* has widespread distribution in a large number of countries. It was found in 17.3% of the overall publications and 45.2% of the publications concerning parasites.

#### 3.2.2. Bacteria

##### *Anaplasma* 

The genus *Anaplasma* includes intracellular Gram-negative bacteria belonging to the family *Anaplasmataceae* from the Rickettsiales order. Most of these bacteria are zoonotic and have a high impact on veterinary health; for instance, *Anaplasma marginale*, *Anaplasma bovis* and *Anaplasma ovis*; and also on human health; for instance, *Anaplasma phagocytophilum* and *Anaplasma platys* [241,242]. The most common species is *A. platys*, responsible for canine cyclic thrombocytopenia, which was found in 11 countries out of 20 in the Mediterranean Basin (Algeria, Croatia, Cyprus, Egypt, Greece, Italy, Morocco, Palestine, Spain, Tunisia and Turkey). Six different tick species were found to be positive and to transmit this pathogen, with Rhipicephalus sanguineus s.l. as the main tick species, and with the highest prevalence in Morocco at 6.25%. The most common host was the dog, with a prevalence of 40.8% and 33% positive blood samples in Italy [72,113,148]. The second most common species is *A. phagocytophilum*, the causative agent of human granulocytic anaplasmosis, found in 10 countries (Algeria, Croatia, Egypt, France, Greece, Italy, Morocco, Spain, Tunisia and Turkey). Out of six tick species found to be positive for this bacterium, *Rh. sanguineus* s.l. was the main tick species, especially on dogs. The highest prevalence of *A. phagocytophilum* in *Rh. sanguineus* s.l. was 13.7% in Egypt. The main pathogen host was cattle, with a prevalence of 40.6% in blood samples in Algeria [104,130].

As seen above, the *Anaplasma* genus is both highly represented in the Mediterranean Basin and also highly screened, with detection in more than 25.8% of the publications analyzed and in 44.9% of the publications concerning bacteria. This can be explained by the number of countries (12) and hosts affected (11), and shows the public health and veterinary importance of this genus in the countries of the Mediterranean Basin, especially for the two zoonotic species *A. phagocytophilum* and *A. platys*.

##### *Bartonella* 

*Bartonella* are Gram-negative bacteria belonging to the family *Bartonellaceae* from the Rhizobiales order, and half of them are known to be zoonotic. They are responsible for diseases such as trench fever and cat-scratch disease, and frequently cause endocarditis [243]. The most commonly detected species in domestic animals is *Bartonella henselae*, responsible for cat-scratch disease, detected in five countries (Algeria, Cyprus, Greece, Italy, and Spain). *Bartonella henselae* were mainly found in *Ixodes ricinus*, with the highest prevalence in Italy at 5.4% [154]. The ticks reported positive were mainly collected on cats. The host reported with the highest prevalence was the cat, with 83.5% in Italy [151]. The other four *Bartonella* species detected were observed in only one or two countries, mainly Italy and Spain.

The *Bartonella* genus is less often screened for and less often found than the previous genus, featuring in only 7.7% of the publications and 13.5% of the publications concerning bacteria. It is mainly screened for in pets (cats and dogs). This genus is mainly transmitted by biting flies and by fleas; however, it is evident that ticks can also be involved [244]. This shows both unequal presence and unequal screening for the *Bartonella* genus in the countries of the Mediterranean Basin. Veterinary interest in the genus focuses on pets.

##### *Borrelia* 

The *Borrelia* genus includes spirochetes bacteria belonging to the family *Spirochaetaceae*. It is divided into two groups: the Lyme borreliosis group responsible for Lyme disease, which is mainly caused by bacteria of the *Borrelia burgdorferi sensu lato* group, and the relapsing fever group, which includes *Borrelia miyamotoi* [245]. The main species found in our study were from the *B. burgdorferi sensu lato* group, with the highest prevalence in animal hosts at 1.47% in dogs from Italy; and in ticks at 53% and 26% in *Rh. sanguineus* s.l. and *Rh. annulatus*, respectively, from cattle in Italy. Concerning *B. afzeeli*, *B. garinii* and *B. valaisiana*, the highest occurrence rates in ticks were 4.3%, 4.3% and 6.4%, respectively, from *Ixodes ricinus* collected from dogs in Spain [51,53,60,127]. The last species detected in the Mediterranean Basin from domestic animals was *Borrelia theileri*, which is a relapsing fever bacterium responsible for bovine borreliosis [246]. It was detected in goats and sheep with 10.8% and 5.8% prevalence, respectively in Algeria [66].

*Borrelia* found in domestic animals from the Mediterranean Basin were mainly from the Lyme Borreliosis group, except for *Bo. theileri*. They were detected in only 4.1% of the publications analyzed and in 7.1% of the publications concerning bacteria. Among these publications, they were more commonly found in ticks (72.7%) than in animal hosts (27.8%). On the basis of these results, research seems to be more focused on the possible circulation of infested ticks than on the potential role of domestic animals as reservoirs of the pathogen. Borrelia is either poorly screened or not common in domestic animals from countries of the Mediterranean Basin.

##### *Chlamydia/Chlamydophila* 

The genera *Chlamydia* and *Chlamydophila* belong to the *Chlamydiales*, which are Gram-negative bacteria responsible for a wide range of diseases throughout almost the entire animal realm [160]. Recently, a few studies carried out in Italy showed that ticks could be vectors of *Chlamydia*/*Chlamydophila* [154,160]. Two bacterial species have been found. The first is *Chlamydia abortus*, from the genus *Chlamydia*, with the highest prevalence at 40.5% in *Ixodes ricinus* from cats in Italy. The second, belonging to the genus *Chlamydophila*, is *Chlamydia psittaci*, with the highest prevalence at 4.4% in *Rh. sanguineus* s.l. from dogs and breeding animals in Italy. No bacteria were found in animal hosts. The *Chlamydia* genus was found in 0.7% of the overall publications and in 1.3% of the publications concerning bacteria. This shows a possible role of ticks in the circulation of *Chlamydia* species; however, transmission and circulation in the domestic animal population in the countries of the Mediterranean Rim cannot be confirmed.

##### *Coxiella* 

Only one pathogen is representative of this genus: *Coxiella burnetii*, responsible for Q fever, which is transmitted by ticks and affects both humans and animals. This disease has a worldwide distribution and can cause febrile illness, endocarditis, meningoencephalitis or pneumonia in humans, while it is mainly asymptomatic in animals apart from sporadic cases of abortion in pregnant animals [247]. It has been found in nine countries of the Mediterranean Basin (Algeria, Cyprus, Egypt, Greece, Italy, Montenegro, Slovenia, Spain and Tunisia), with the highest prevalence in animals at 71.2% from camels in Algeria and 10.2% in *Rh. sanguineus* s.l. from goats and sheep in Cyprus [163,177]. This genus was found in 8.9% of the publications and in 15.4% of the publications concerning bacteria. For *C. burnetii*, most of the publications investigated the ticks as well as their hosts, indicating a level of public health and veterinary interest.

##### *Ehrlichia* 

*Ehrlichia* spp. is a genus closely related to *Anaplasma* spp., and is responsible for human monocytotropic ehrlichiosis (*Ehrlichia chaffeensis* and *Ehrlichia canis*) and for canine ehrlichiosis (*Ehrlichia canis*) [248]. The main species in domestic animals from the Mediterranean Basin is *Ehrlichia canis*, which is responsible for disease in both humans and dogs. It has been detected in 10 countries (Algeria, Croatia, Cyprus, Egypt, France, Greece, Italy, Palestine, Spain, and Turkey) and its highest prevalence was found at 48.5% in dogs from Italy and 6.6% in *Rh. sanguineus* s.l. collected from dogs in Turkey [123,206]. The other four species found in our review, *E. equi*, *E. ewingii*, *E. minacensis* and *Candidatus* E. urmitei, have a minor health impact and were each found in only one country. The *Ehrlichia* genus was the third most common bacterial genus found or screened for after *Anaplasma* and *Rickettsia*, featuring in 11.8% of the overall publications and 20.5% of the publications concerning bacteria. Among the publications dealing with the *Ehrlichia* genus, 93.5% concerned *E. canis*.

##### *Neoehrlichia* 

Of the four bacteria in this genus, only one is a human pathogen: *Neoehrlichia mikurensis*, which causes chronic lymphocytic leukemia, for example. Its vectors are from the *Ixodes* genus, and rodents are the most well-known hosts [249]. In domestic animals, it has been detected only in Spain with 1% prevalence in *I. ricinus* from cattle [136]. This species was rarely found or poorly screened for, featuring in only 0.4% of the publications and 0.6% of the publications concerning bacteria.

##### *Francisella* 

Bacteria from the genus *Francisella* are Gram-negative bacteria with one important species, *Francisella tularensis*, responsible for tularemia in humans and animals [250]. In the domestic animals of the Mediterranean Basin, only the genus *Francisella* spp. level was identified. It was found only in Italy, with a prevalence of 66%, 21% and 8% in *Hyalomma marginatum*, *Rh. bursa* and *Rh. (Boophilus) annulatus* from cattle, respectively. As with the *Neoehrlichia* genus, no *Francisella* was detected directly in animal hosts [149].

##### *Leptospira* 

The genus *Leptospira* includes spirochetes and zoonotic bacteria that are responsible for leptospirosis worldwide. The first case was documented over 100 years ago. The bacteria are usually transmitted by direct or indirect contact with a contaminated element, but can also be found in ticks [251,252]. Again, only the genus *Leptospira* spp. was detected. It was found in Egypt with a prevalence of 50%, 41%, 40% and 29% from camel, sheep, cattle and buffalo, respectively [184]. Unlike the two previous genera, *Leptospira* spp. was only found in domestic animals and not in ticks. However, similarly to the two previous genera, it was found in 0.4% of the overall publications and in 0.6% of publications concerning bacteria. Nevertheless, the *Leptospira* genus has already been found in ticks in Europe [252], but this was not found to be reported in the present review.

##### *Mycoplasma* 

The genus *Mycoplasma* is composed of commensal and pathogenic bacteria that can cause anemia in a wide range of mammals [253,254,255]. *Mycoplasma haemocanis*, *My. haemofelis*, *Candidatus* My. haemonutum, *Candidatus* My. haematoparvum and *Candidatus* My. turicensis were found, but only *My. heamocanis* and *Candidatus* My. haematoparvum were found in two countries (Greece and Turkey). The prevalence of *My. haemocanis* was 5.6% in dogs from Greece and 26.2% in dogs from Turkey. For *Candidatus* My. haematoparvum, the prevalence was 4.2% from dogs in Greece and 6.7% from dogs in Turkey [185,186]. The other three species were each found in only one country. As with the *Leptospira* genus, the *Mycoplasma* genus was found only in animals in this review. It was found in 2.2% of the publications and in 1.3% of the publications concerning bacteria.

##### *Rickettsia* 

The genus *Rickettsia* is one of the most important tick-borne pathogen genera. It is divided into two groups: the spotted fever group (SFG), including *Rickettsia conorii*, causative agent of Mediterranean spotted fever, and the typhus group (TG), which is less well-known and includes, for example, *Rickettsia typhi* [256,257]. Seventeen species were detected in domestic animals in this study. Sixteen of these were from the SFG (*R. aeschlimannii*, *R. africae*, *R. conorii*, *R. conorii israelensis*, *R. felis*, *R. helvetica*, *R. hoogstraalii*, *R. massiliae*, *R. monacensis*, *R. raoultii*, *R. rickettsia*, *R. rhipicephali*, *R. sibirica mongolotimonae*, *R. slovaca*, *Candidatus* R. barbariae and *Candidatus* R. goldwasserii), and one was from the TG: *R. typhi*. Of these *Rickettsia* spp., *Rickettsia massiliae* (SFG group) was the most widespread bacterium and was detected in 10 countries (Algeria, Cyprus, France, Greece, Israel, Italy, Lebanon, Palestine, Spain and Tunisia). Its highest prevalence was 40.4% in *Rh. sanguineus* s.l. collected from cattle, dogs and sheep from Algeria, and 2.7% from camel blood in Tunisia [175,213]. The second most common bacterium was *R. africae* (SFG group), found in eight countries (Egypt, France, Israel, Italy, Lebanon, Palestine, Tunisia and Turkey), with the highest prevalence at 26.7% in *Hyalomma impeltatum* collected from cattle and camels in Egypt, and 0.3% in camel blood from Tunisia [194,201]. *Rickettsia typhi* was the only bacterium from the TG group, detected with a prevalence of 29.7% from cats in Greece [155]. On the Mediterranean Rim, the genus *Rickettsia* is widespread and diversified in domestic animals and their ticks, and is widely screened; it was found in 25.3% of the publications overall, and in 44.2% of the publications concerning bacteria. This genus is the second most commonly studied after *Anaplasma* spp. However, the SFG group (98.6% of the publications) was of the greatest scientific interest compared to the TG group (1.4% of the publications).

#### 3.2.3. Viruses

##### *Capripoxvirus* 

This genus includes species that affect a broad range of domestic ruminants and that have a considerable economic impact, especially in Africa and the Middle East. It covers three species: lumpy skin disease virus (LSDV), goatpox virus (GTPV) and sheeppox virus (SPV) [258]. Among these three species, only LSDV was found in ticks from domestic animals in this review. It was found in ticks from the genera *Amblyomma*, *Hyalomma* and *Rhipicephalus* from cattle in Egypt, with a prevalence of 65.5% [224]. It was the least commonly found virus (0.4% of the overall publications and 7.2% of the publications concerning viruses). This genus does not represent a major threat in veterinary health in the countries of the Mediterranean Rim. Nonetheless, it should still be considered a potential threat, considering the range of ticks in which it has been found.

##### *Flavivirus* 

The flaviviruses are an important part of the arboviruses. They are transmitted by both mosquitoes and ticks. They mainly infect mammals and account for a large proportion of the recent outbreaks of public health and veterinary concern in terms of morbidity and mortality [259]. The most well-known tick-borne *flavivirus*, tick-borne encephalitis virus (TBEV), was found in two countries in domestic animals: in Greece, with a prevalence of 1.4% from *I. ricinus* ticks from goats, and in Italy, with a prevalence of 16.43% in goats [224,225]. TBEV was the second most common virus species, found in a total of 0.7% of publications and 14.3% of the publications concerning viruses.

##### *Orthonairovirus* 

Globally, this genus is mainly represented by one species: Crimean–Congo hemorrhagic fever virus (CCHFV). This virus is transmitted by ticks, mainly from the genus *Hyalomma*, causes severe or even fatal human disease across almost all of the Old World, and its range has expanded with climate change [260]. In domestic animals from countries of the Mediterranean Basin, the virus has been found in five different countries (Albania, Greece, Egypt, Spain, and Turkey). The highest rates of occurrence were 90% from sheep in Albania and 6.88% from three tick genera (*Hyalomma* spp., *Rhipicephalus* spp. and *Ixodes* spp.) collected from buffalo, cattle, goats and sheep in Turkey [226,230]. CCHFV was the most commonly screened or found virus, featuring in about 78.5% of the publications concerning viruses and in 4.1% of the overall publications. In order to determine the expansion of the virus, numerous research studies have been performed on the tick species that are potential vectors of the virus.

### 3.3. Ticks Positive for Tick-Borne Pathogens from Domestic Animals in the Mediterranean Basin

**Figure 4:**
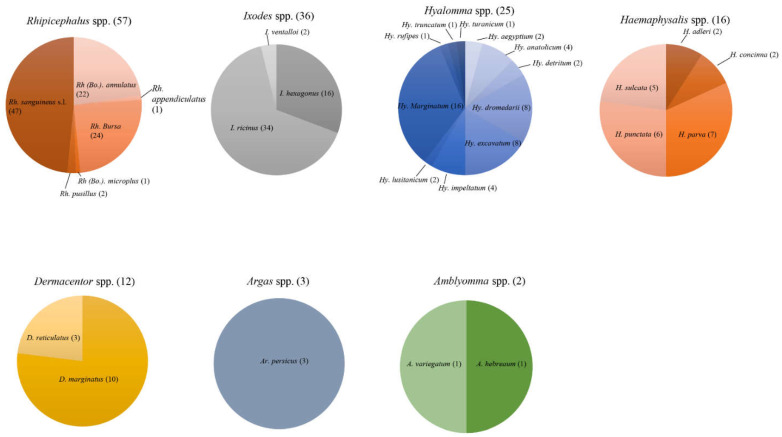
Engorged ticks collected from domestic animals and positive for TBPs (the number of pathogens identified per taxon is indicated in brackets).

In all, 35 tick species from seven genera found on domestic animals in the Mediterranean Basin were positive for TBPs. Out of the seven genera, six belonged to the hard ticks (Ixodidae): *Amblyomma*, *Dermacentor*, *Haemaphysalis*, *Hyalomma*, *Ixodes* and *Rhipicephalus*. These genera are widely distributed in this region. The last genus, which was collected in Algeria and Egypt, represents the soft ticks (Argasidae): *Argas* (Table S1). The diversity of tick species positive for TBPs varies between these genera (Figure 4).

#### 3.3.1. Ixodidae

##### Genus *Rhipicephalus*

The top-ranking genus in terms of TBP diversity detected in the Mediterranean Basin was the genus *Rhipicephalus* (Table S1). It has a worldwide distribution and is very broadly present in the Mediterranean Basin, where it can target a large range of wild and domestic animals [261,262]. This genus transmits pathogens that cause diseases with global health impacts, such as anaplasmosis, babesiosis, ehrlichiosis and rickettsiosis [261,263]. However, only six species were identified for the detection of 57 pathogenic species in a total of 13 countries: *Rh (Bo.). annulatus*, *Rh. appendiculatus*, *Rh. bursa*, *Rh (Bo.). microplus*, *Rh. pusillus* and *Rh. sanguineus* s.l. Out of these 57 TBP species, 47 were detected in *Rh. sanguineus* s.l., which was the tick species with the highest number of pathogenic species found in our review (Figure 4). It is a tick species known for the transmission of a number of TBPs, such as *E. canis*, *R. conorii* and *B. canis*, f-ound in our review. This tick species is highly present in domestic animals and has a tropism for pets, such as dogs, as well as livestock, such as cattle [261]. This tropism and the TBPs transmitted by this species could be the reasons underlying both the scientific interest in this species and the number of TBPs found in domestic animals.

##### Genus *Ixodes*

The second-ranking genus in terms of pathogen diversity was the genus *Ixodes* (Table S1). This genus is mainly known for the transmission of Lyme disease. The ticks are present mainly in Europe, where due to their pleiotropic feeding, they are responsible for the spread of numerous TBPs [262]. They target a broad range of hosts (ubiquity) and have a high impact on human and animal health [261,263]. Only three species were detected in countries of the Mediterranean Basin: *I. hexagonus*, *I. ricinus* and *I. ventalloi*. A total of 36 pathogens were found on six animal hosts. This genus has the highest number of pathogens detected for the lowest number of tick species, with a total of 36 pathogen species for just three tick species: *I. hexagonus*, *I. ricinus* and *I. ventalloi*. The main species is *I. ricinus*, in which 34 TBPs were found out of the 36 found in the genus (Figure 4). This species is ubiquitous and can be found on mammals, reptiles and avifauna. *Ixodes ricinus* is mainly known for the transmission of *B. burgdorferi* s.l., the causative agent of Lyme disease. However, as shown in our review, it can be positive for a wide range of pathogens. This could be due to the large array of hosts on which this species can feed.

##### Genus *Hyalomma*

The genus *Hyalomma* ranks third in terms of TBPs detected. It is known for the transmission of CCHFV and of bacteria such as *Rickettsiae*. The ticks are large in size (5–6 mm) with tropism for large mammals upon reaching their adult forms, and for small mammals and birds while in their immature stages. *Hyalomma* species are mainly distributed in the southern part of the Mediterranean Basin, with a slow increase in range in Western Europe and the Balkans [261,263]. In the Mediterranean Basin, a total of 11 species were found, which is the highest number of species among all the tick genera. They were found on nine hosts in 12 countries and were found to be infected by a total of 25 TBPs (Table S1). The number of hosts, countries and pathogens associated with this genus indicates its public health importance and the considerable threat it poses. Among the 11 species of the genus *Hyalomma*, the species found positive for the largest number of TBPs was *Hy. marginatum* (Figure 4). Out of 25 species found in the genus, a total of 16 TBPs were found. Concerning domestic animals, *Hy. marginatum* affects a large range of livestock and can be responsible for TBDs of veterinary importance, such as infections with *Rickettsia* spp., CCHFV, *Babesia* spp. and *Theileria* spp. These characteristics, along with the tropism of the immature-stage ticks for birds, could underpin the public health importance of this species.

##### Genus *Haemaphysalis*

The genus *Haemaphysalis* is the fourth most common genus in terms of pathogens detected (Table S1). The ticks are small in size and target mammals upon reaching their adult stage, and a wide array of hosts when still in their immature stages. They are known to have veterinary importance in livestock and are present in Asia [263]. On domestic animals from the Mediterranean Basin, the genus *Haemaphysalis* was detected in seven different countries, and ticks were positive for 16 TBPs. Five different species were found: *H. adleris*, *H. concinna*, *H. parva*, *H. punctata* and *H. sulcata*. Among these, the species found to be positive for the highest number of TBPs was *H. parva* (Figure 4). This species is mainly distributed in the Mediterranean Basin, with key tropism for domestic ungulates when the ticks are in the adult stage. It can transmit a wide range of TBPs, such as *Rickettsia* spp., *C. burnetii* and *F. tularensis* [264]. Unlike the genus *Hyalomma*, the pathogen distribution in the different species of *Haemaphysalis* is more uniform, especially between *H. parva*, *H. punctata* and *H. sulcata*. This could explain the overall interest in the genus *Haemaphysalis* on domestic animals across the countries of the Mediterranean Basin.

##### Genus *Dermacentor*

The genus *Dermacentor* ranked fifth in terms of TBPs detected (Table S1). Similarly to the genus *Ixodes*, it is mainly present in Europe, where it transmits a broad range of TBPs [261,262]. The genus is represented by two common species in the Mediterranean Basin: *D. marginatus* and *D. reticulatus*. They transmit a wide array of pathogens, including *Rickettsia* spp. and *Babesia* spp., which are of veterinary and human health importance. All the development stages were found in six different domestic animals from eight different countries. Twelve pathogen species have been identified in the two tick species. The main species was *D. marginatus*, which was found to be positive for 9 TBPs (Figure 4). This species is known for its tropism on wild and domestic ungulates, but also for transmission of *B. canis* and *R. slovaca* [261].

##### Genus *Amblyomma*

The genus *Amblyomma* was the last-ranking genus of hard ticks in terms of TBPs detected (Table S1). It can be found in nearly all terrestrial animals and occurs mainly in the tropical and sub-tropical areas of Asia, Africa and Oceania [265]. Recently, it appeared in the Mediterranean Basin, for instance in Corsica [199,266]. Due to the geographical distribution of this genus, it was rarely found in the Mediterranean Basin and only two species (*A. hebreaum* and *A. variegatum*) have been identified. Positive ticks were only found on cattle. The virus and bacteria detected were lumpy skin disease virus and *Rickettsia africae* (Table 2, Figure 4).

#### 3.3.2. Argasidae

Among the soft ticks, only one species was found: *Argas persicus*. This species is mainly found on domestic birds. It is mainly found in the Mediterranean Basin but is primarily involved in the transmission of bird-related pathogens [261,263,267]. In our review, it was found on two hosts, and three pathogen species were found (Table S1, Figure 4). This genus is one of the less commonly collected ticks in the countries of the Mediterranean Basin, along with the genus *Amblyomma* of the hard ticks group.

### 3.4. Domestic Animal Hosts of Both Positive Ticks and TBPs in the Countries of the Mediterranean Basin

All the information in this part brings together data about domestic animals infested by TBPs and about domestic animal hosts of positive engorged ticks. However, the data about positive engorged ticks do not confirm the vector character of the ticks for the different TBPs. The domestic animals from countries of the Mediterranean Basin identified in this review can be divided into two groups: livestock animals and pets.

Livestock was composed of nine species (Table 2), which were reasonably well studied in the literature under review. It appears that cattle, sheep and goats were studied in more than 13 countries and were the subject of the highest number of studies. This can be explained by the wide range of tick-borne pathogen genera targeting these species (Table 2), and the economic importance of livestock in the countries of this region [268]. Among the 90 TBPs identified in this review, about 59% were found in positive ticks from cattle, with more than 40% found directly on the animal. On the other hand, some species (buffalo, chickens, donkeys and pigs) were well studied, and available data were found in only one or two countries. The number of TBPs detected in these animals or in their ticks is much lower (Table 2).

The pet group was composed of only three species: cats, dogs and rabbits. Among these three species, the dog was clearly the more commonly studied pet. Available data about the detection of TBPs in dogs (77 studies) and their ticks (63 studies) were found for 14 countries, while cat and rabbit data were found in only five countries and one country, respectively (Table 2).

Among the 90 TBPs listed in this review (in livestock or pets), 66 were detected in ticks from animals and 69 directly in the animals. The highest number of pathogens was found in ticks on dogs (66.7%) (Table 2). The list of TBPs reported in each domestic animal host and their ticks is summarized in the Table S2.

**Figure 5:**
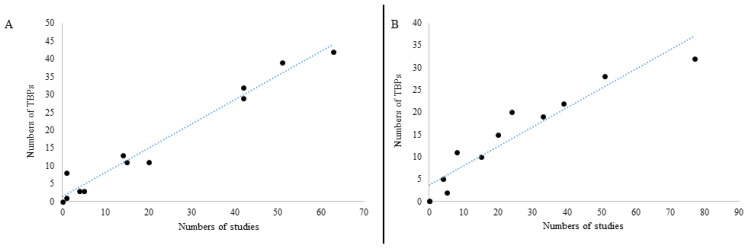
ScatterPlot diagrams showing a strong linear correlation between (**A**) the number of studies and the number of TBPs from positive ticks by domestic animal (correlation coefficient R = 0.98), and (**B**) between the number of studies and the number of TBPs by positive domestic animal (correlation coefficient R = 0.95).

The research effort, expressed by the number of publications, varies greatly from one animal to another. This is why it is difficult to conclude that an animal, or its ticks, are more or less susceptible to being infected by TBPs. In this sense, Figure 5 shows a positive correlation between the number of studies and the number of TBPs found in animals (Figure 5B) or in their ticks (Figure 5A). The very high correlation coefficient (0.95 and 0.98, respectively) expresses a strong correlation between these variables. If we assume that correlation does not imply causation, we can nevertheless observe a correlation between the number of TBPs found and the number of publications focusing on the different domestic animal species, and conclude what may be obvious: the more we seek, the more we find.

### 3.5. Biogeography, Diversity and Distribution in the Mediterranean Basin

#### 3.5.1. Overall Analysis of the Four Main Regions in the Mediterranean Basin

In this section, we divide the countries of the Mediterranean Basin into four different areas to allow for better comparison of the data found. Western Europe is made up of France, Italy, Malta, Monaco and Spain, which represented 45.7% of the publications. North Africa, which includes Algeria, Egypt, Libya, Morocco and Tunisia, had a percentage of 26.1% of the total publications included in this review. The Middle East, which is composed of Cyprus, Israel, Lebanon, Palestine, Syria and Turkey, represented 17.7%. The Balkans area is made up of Albania, Bosnia-Herzegovina, Croatia, Greece, Montenegro and Slovenia, and had 10.33% of the publications. The five countries in which no pathogens were found on domestic animals or their ticks were Bosnia-Herzegovina, Libya, Malta, Monaco and Syria.

##### Western Europe

**Figure 6:**
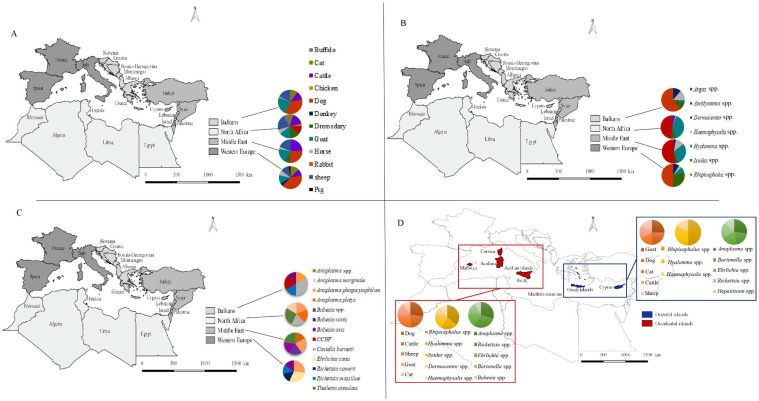
Map of the main domestic animal hosts (**A**), main positive tick genera (**B**), main TBPs in the four areas of the Mediterranean Basin (**C**), and main domestic animal hosts, positive tick genera and TBP genera in the western and eastern islands (**D**).

In the five countries of this area, tick-borne pathogens on domestic animals were detected in Italy, France and Spain. These are also the three countries with the highest number of publications in the Mediterranean Basin. A total of eight domestic animal species were found to be carriers of positive ticks and pathogens: cats, cattle, dogs, donkeys, goats, horses, sheep and pigs. From our data, six tick genera from domestic animals were found to be positive for TBPs: *Amblyomma* spp., *Dermacentor* spp., *Haemaphysalis* spp., *Hyalomma* spp., *Ixodes* spp. and *Rhipicephalus* spp. The most commonly found host was the dog (Figure 6A), which was the main target of the genus *Rhipicephalus.* In addition, the most predominant tick genus found out of the seven genera collected was *Rhipicephalus* spp. (Figure 6B), a vector of pathogens, such as *B. canis* and *E. canis*. The other main tick genera were *Ixodes*, a ubiquitous genus, and *Hyalomma*, mainly found on livestock. Of 68 pathogens detected in this region, five were included in nearly 60% of the publications in Western Europe. They belonged to the bacteria and parasite groups *E. canis*, *A. phagocytophilum*, *R. conorii*, *B. canis* and *R. massiliae* (Figure 6C). It appears that the papers found in Western Europe mainly focused on dogs, which could explain the high percentage of *Rhipicephalus* ticks found and TBPs related to dogs. This focus can be explained by the central place of the dog in human activities, as pets, hunting dogs or stray dogs.

##### North Africa

The North Africa area has the largest diversity of domestic animal hosts of pathogens and positive ticks reported in the countries of the Mediterranean Basin, with a total of 10 domestic animal hosts: cats, cattle, chickens, buffalo, dogs, dromedaries, goats, horses, rabbits and sheep. The main animals reported were from the livestock group: cattle, sheep, goats and dromedaries (Figure 6A). From these hosts, five tick genera were found to be positive for TBPs: *Argas* spp., *Haemaphysalis* spp., *Hyalomma* spp., *Ixodes* spp. and *Rhipicephalus* spp. Even though the main tick genus reported was the same as in Western Europe (genus *Rhipicephalus*), it was found both in livestock and pets, with equal importance in terms of health. The other main tick genus was *Hyalomma* spp. which was mainly found on livestock (Figure 6B). The main TBPs reported belong to the bacteria and parasite groups: *A. marginale*, *A. phagocytophilum*, *A. platys*, *C. burnetii* and *T. annulata* (Figure 6C). They are mainly related to livestock rather than pets. In North Africa, research seems to be more focused on livestock veterinary health monitoring compared to Western Europe.

##### The Middle East

In this area, a total of six domestic animals were reported to be carriers of positive ticks and TBPs: cattle, dogs, dromedaries, goats, horses and sheep. Similarly to Western Europe, the main domestic animal host found was the dog, closely followed by cattle, featuring in 66.7% and 62.5% of the total publications in the area, respectively (Figure 6A). From the domestic animal hosts, five tick genera were reported to be positive for TBPs: *Dermacentor* spp., *Haemaphysalis* spp., *Hyalomma* spp., *Ixodes* spp. and *Rhipicephalus* spp. As with the two previous areas, *Rhipicephalus*, which can be found both on livestock and pets, was the main tick genus found. The other main tick genera were *Hyalomma* and *Haemaphysalis*, which also infested livestock (Figure 6B). Of the 45 pathogens found in the Middle East, the most predominant were *Anaplasma* spp., *A. phagocytophilum*, *Babesia* spp., *B. ovis* and *T. annulata*. They were identified in 62.5% of the publications in the area (Figure 6C). In the Middle East, the data available were related to the veterinary health of both pets and livestock to equal extents. However, they were mainly from Turkey (75.5%).

##### The Balkans

A total of six domestic animal hosts were reported to be carriers of positive ticks and TBPs: cats, cattle, dogs, goats, horses and sheep. Of the six domestic animal hosts reported, the main host was the dog, followed by the goat, which both seemed to be the focus of veterinary research in the four areas, probably due to their economic and social importance (Figure 6A). Five tick genera were reported to be positive for TBPs: *Dermacentor* spp., *Haemaphysalis* spp., *Hyalomma* spp., *Ixodes* spp. and *Rhipicephalus* spp. Of the five genera found in the Balkans, the main positive tick genus collected on domestic animals was *Rhipicephalus* spp., followed by the genera *Haemaphysalis*, *Ixodes* and *Dermacentor* (Figure 6B). A total of 32 pathogens were found in the seven countries of the Balkans area. The most frequently detected were the bacteria *C. burnetii*, *A. platys* and *R. massiliae*, the parasite *B. canis* and the CCHF virus (Figure 6C). These were reported in 90% of the publications. As for the Middle East, these TBPs can be found in livestock and pets.

#### 3.5.2. Focus on Insular Tick-Borne Pathogens in Domestic Animals and Their Ticks

The Mediterranean islands are an important place for animal migrations, human activities and pathogen circulation due to their geographical situation. A total of 44 publications were reported on islands, representing 16% of the publications taken into account in this review. These 44 publications were considered separately from the previous dataset. Out of these 44 publications, most came from the largest islands of the Mediterranean Basin: Sardinia, Sicily, Corsica and Cyprus, while some data were reported from smaller islands, such as the Greeks islands. We decided to split the islands of the western Basin, including the Aeolian islands, Corsica, Sardinia, Sicily and Mallorca (36 publications), from the eastern Basin, including Crete, Cyprus, Ios, Mykonos, Santorini, Skiathos, Skopelos and Tinos (8 publications) (Table 3).

On the islands, a total of nine domestic animal hosts were studied: cats, cattle, dogs, donkeys, goats, horses, pigs, rabbits and sheep. Despite this host diversity, the majority of the islands followed the same schema as in the rest of the Mediterranean Basin, with the dog predominating as host of positive ticks and TBPs (43.2% of the island publications), followed by cattle, sheep and goats. We also observed a higher diversity of domestic animal hosts (eight species) in the data reported in the western islands (cats, cattle, dogs, donkeys, goats, horses, pigs and sheep) than in the eastern islands (five species: cats, cattle, dogs, goats and sheep) (Table 3). In the western islands, most of the publications found were about dogs (44.4%), followed by cattle and sheep (each featuring in 36.1% of the publications). In the eastern islands, the main domestic animal hosts reported were goats and dogs, each featuring in 37.5% of the publications (Figure 6D).

A total of six hard tick genera, and no soft ticks, were found to be positive in the data from the islands: *Amblyomma* spp., *Dermacentor* spp., *Haemaphysalis* spp., *Hyalomma* spp., *Ixodes* spp. and *Rhipicephalus* spp. (Table 3). Of these genera, the main positive tick genus reported was *Rhipicephalus* spp., featuring in 43.2% of the island publications. This follows the same schema as the overall data. Nevertheless, the second most common genus reported was *Hyalomma*. This corresponds to the observations made in North Africa and the Middle East, and differs from Western Europe and the Balkans. This finding could be due to the wide distribution of *Hyalomma* in the Mediterranean Basin, especially on the south and east borders, with some species also on the north border [261]. This could also testify to the extension of the distribution area of this genus through animal migration and with the facilitation of climate change. This may result in the spread of TBPs, such as the CCHF virus transmitted by these ticks [19,269,270]. In this dissemination, islands may play an important role as places of transition between the different borders of the Mediterranean Basin [271]. This suggests a potential sentinel role of islands when monitoring dissemination of this tick genus and their TBPs on a continental scale (Figure 6D).

In the western islands, TBPs were screened and found in ticks from domestic animals in 63.9% of the papers. Meanwhile, the corresponding value was only 37.5% of the publications in the eastern islands. For both groups of islands, the main positive tick genus found was *Rhipicephalus* spp., in 69.6% and 100% of the publications, respectively. This shows the importance of this genus on islands across the whole Mediterranean Basin. For both western and eastern islands, this genus was succeeded in prevalence by the genus *Hyalomma* (Figure 6D). From the data analyzed, it seems that there was a stronger focus on TBPs in ticks from domestic animals in the western islands than in the eastern islands.

Of the 18 genera of pathogens reported in the data, 14 genera were reported in the islands, representing 77.8% of the total genera (Table 3). The main TBP genera detected were *Anaplasma* spp. and *Rickettsia* spp., reported in 43.2% and 40.9% of the publications concerning islands, respectively. This follows the same schema as the overall data. In the western islands, the 14 TBP genera were reported (66.7% bacteria and 33.3% parasites), while only nine were reported (77.8% bacteria and 22.2% parasites) in the eastern islands. In both groups, the main reported genus was *Anaplasma*, followed by *Rickettsia* (Figure 6D). They were both reported in 41.7% of the western island publications, and in 50% and 37.5% of the eastern island publications, respectively. No viruses were reported in either of the island groups.

Even though few data were reported for the islands (16%), a high diversity of TBPs was reported. This may indicate the importance of islands in the monitoring of TBPs in domestic animals in the Mediterranean Basin.

## 4. Conclusions

In this study, 90 TBPs from 18 genera of bacteria, parasites and viruses were reported in domestic animals and their ticks in the countries of the Mediterranean Basin. Most pathogens were bacteria, followed by parasites and viruses. The main genera detected were bacteria: *Anaplasma* spp. and *Rickettsia* spp. The data collected reflected a high diversity of TBPs in domestic animals and their ticks in the countries of the Mediterranean Basin, which shows their importance in veterinary and human health. A wide range of pathogens was reported in seven positive tick genera (six hard ticks and one soft tick) and 31 tick species. The most-reported genus was *Rhipicephalus* spp., a genus found in a large range of domestic animals, from livestock to pets. This genus is also known for the transmission of TBPs from the genera *Anaplasma* and *Rickettsia*. These TBPs and positive tick genera were reported in 12 different domestic animal hosts divided into two groups: livestock and pets. The main domestic animal hosts were both dogs and cattle, from which the highest diversity of TBPs was reported. This seems linked to the quantity of data reported for these hosts. In the four areas of the Mediterranean Basin (Western Europe, North Africa, the Middle East and the Balkans), the main studied host was the dog (except in North Africa) and the main positive tick genus was *Rhipicephalus*. Depending on the area, the second most important genera were *Ixodes* or *Hyalomma*. This could be due to the distribution area of *Ixodes* spp. in Europe and the distribution area of *Hyalomma* spp. on the south border of the Mediterranean Basin and its expansion through the islands [260,267]. The diversity of the TBPs identified in this review was linked to the domestic animals targeted in the studies and to the animals’ veterinary and social importance. In all, 16% of the publications concerned TBPs from domestic animals and their ticks on islands, but high diversity of domestic animal hosts (nine of 12), positive tick genera (six of seven) and TBP genera (14 of 18) was reported for the islands. This shows the importance of the Mediterranean islands in the monitoring of TBPs in this region as sentinel territories. The development of research on the islands could provide a better understanding of their role as a hotspot for the circulation of ticks and tick-borne pathogens.

## Figures and Tables

**Table 1:** Tick-borne pathogens detected in domestic animals or engorged ticks found on domestic animals in the Mediterranean Basin.

Pathogen	Zoonotic (Yes/No)	Engorged Positive Ticks Collected from Hosts	Positive Tick Hosts	Positive Pathogen Hosts	Countries	References
**Parasites: Nematoda**						
*Cercopithifilaria*						
*Cercopithifilaria bainae*	No	*Rhipicephalus sanguineus* s.l.	Dog	Dog	Greece, Italy	[22,23]
**Parasites: Apicomplexa**						
*Babesia*						
*Babesia* spp.		*Hyalomma excavatum*, *Hyalomma marginatum*, *Rhipicephalus sanguineus* s.l.	Cattle	Cattle, Dog, Donkey, Goats, Pigs, Sheep	Egypt, Italy, Morocco, Turkey	[24,25,26,27,28,29,30,31,32,33]
*Babesia bigemina*	No	*Hyalomma marginatum*, *Rhipicephalus (B) annulatus*, *Rhipicephalus bursa*, *Rhipicephalus sanguineus* s.l.	Cattle, Dog, Sheep	Cattle, Pig, Sheep	Algeria, Egypt, Italy, Turkey	[29,32,33,34,35,36,37,38,39,40,41,42]
*Babesia bovis*	Yes	*Dermacentor marginatus*, *Hyalomma marginatum*, *Ixodes ricinus*, *Rhipicephalus sanguineus* s.l.	Cattle, Goat, Sheep	Buffalo, Cattle	Algeria, Egypt, Italy, Turkey	[29,30,32,34,35,39,40,41,43,44]
*Babesia caballi*	No	*Rhipicephalus (B) annulatus*	Cattle	Dog, Donkey, Horse	Croatia, France, Italy,	[32,42,45,46,47]
*Babesia canis*	No	*Dermacentor reticulatus*, *Ixodes hexagonus*, *Rhipicephalus sanguineus* s.l.	Dog	Dog	Croatia, France, Italy, Spain, Turkey	[27,32,37,41,45,48,49,50,51,52,53,54,55,56,57,58,59,60]
*Babesia capreoli*	No	*Ixodes hexagonus*, *Ixodes ricinus*, *Rhipicephalus sanguineus* s.l.	Dog	data not found	Italy	[60]
*Babesia divergens*	Yes	*Ixodes ricinus*, *Rhipicephalus sanguineus* s.l.	Dog	Cattle	France, Italy	[60,61,62]
*Babesia equi*	No	data not found	data not found	Horse	Italy	[63]
*Babesia gibsoni*	No	*Dermacentor reticulatus*, *Ixodes hexagonus*, *Ixodes ricinus*, *Rhipicephalus sanguineus* s.l.	Dog	Dog	Croatia, Italy, Spain	[45,48,51,59]
*Babesia major*	No	data not found	data not found	Cattle	France, Turkey	[36,50]
*Babesia microti*	Yes	*Dermacentor marginatus*, *Ixodes hexagonus*, *Ixodes ricinus*, *Rhipicephalus sanguineus* s.l.	Dog, Goat, Sheep	Cat, Dog	Italy, Turkey	[32,37,41,60,64]
*Babesia motasi*	No	data not found	data not found	Sheep	Spain	[65]
*Babesia occultans*	No	data not found	data not found	Cattle	Egypt	[35]
*Babesia ovis*	No	*Dermacentor marginatus*, *Haemaphysalis concinna*, *Haemaphysalis parva*, *Ixodes ricinus*, *Rhipicephalus bursa*, *Rhipicephalus sanguineus* s.l.	Dog, Goat, Sheep	Cattle, Goat, Sheep	Algeria, Italy, Palestine, Spain, Turkey	[25,33,36,37,41,65,66,67,68]
*Babesia venatorum*	Yes	*Ixodes hexagonus*, *Ixodes ricinus*, *Rhipicephalus sanguineus* s.l.	Dog	data not found	Italy	[60]
*Babesia vogeli*	No	*Dermacentor marginatus*, *Ixodes ricinus*, *Rhipicephalus sanguineus* s.l.	Cat, Dog, Goat, Sheep	Dog	Croatia, Cyprus, France, Italy, Palestine, Spain, Turkey	[27,41,45,49,51,56,57,59,67,69,70,71,72,73]
*Hepatozoon*						
*Hepatozoon* spp.		*Rhipicephalus sanguineus* s.l.	Dog	Cat, Dog	Croatia, Cyprus, Italy, Spain, Turkey	[22,23,27,51,52,67,69,72,74,75,76,77]
*Hepatozoon canis*	No	*Haemaphysalis parva*, *Ixodes hexagonus*, *Ixodes ricinus*, *Rhipicephalus sanguineus* s.l.	Dog	Cat, Dog	Croatia, Cyprus, France, Greece, Italy, Palestine, Spain, Turkey	[22,23,27,50,51,52,67,69,72,75,77,78,79]
*Hepatozoon felis*	No	*Haemaphysalis concinna*, *Rhipicephalus sanguineus* s.l.	Dog	Cat	Greece, Italy, Turkey	[22,75,80]
*Theileria*						
*Theileria* spp.		*Hyalomma anatolicum*, *Hyalomma dromedarii*, *Rhipicephalus sanguineus* s.l.	Cattle, Dromedary	Cat, Cattle, Dogs, Donkey, Goat, Pigs, Sheep	Algeria, Egypt, Italy, Spain, Turkey	[26,30,32,51,65,68,81,82,83,84,85]
*Theileria annae*	No	*Dermacentor reticulatus*, *Ixodes hexagonus*, *Ixodes ricinus*, *Rhipicephalus sanguineus* s.l.	Dog	Cattle, Dog, Donkey, Horse	Croatia, France, Italy, Spain	[29,45,47,48,56,86]
*Theileria annulata*	No	*Hyalomma anatolicum*, *Hyalomma detritum*, *Hyalomma dromedarii*, *Hyalomma excavatum*, *Rhipicephalus (B) annulatus*	Cattle, Dromedary, Sheep	Buffalo, Cattle, Dromedary, Donkey, Goat, Pig, Sheep	Algeria, Egypt, Italy, Spain, Tunisia, Turkey	[28,32,33,34,35,36,37,40,44,83,84,86,87,88,89,90,91,92,93,94,95]
*Theileria buffeli*	No	*Dermacentor marginatus*, *Haemaphysalis punctata*, *Hyalomma marginatum*, *Ixodes hexagonus*, *Ixodes ricinus*, *Rhipicephalus (B) annulatus*, *Rhipicephalus bursa*, *Rhipicephalus sanguineus* s.l.	Cat, Cattle, Dog, Goat	Cattle, Horse	Algeria, France, Italy, Spain, Turkey	[36,38,42,44,50,60,86]
*Theileria cervi*	No	*Ixodes ricinus*, *Rhipicephalus sanguineus* s.l.	Dog	Data not found	Italy	[60]
*Theileria equi*	No	*Hyalomma marginatum*, *Ixodes ricinus*, *Rhipicephalus (B) annulatus*, *Rhipicephalus sanguineus* s.l.	Cattle, Dog, Horse	Cattle, Dog, Donkey, Horse	Algeria, Croatia, Italy, Spain	[32,38,41,42,44,45,47,51,63,78,96]
*Theileria lestoquardi*	No	data not found	data not found	Buffalo, Sheep	Egypt, Turkey	[82,89]
*Theileria luwenshuni*	No	data not found	data not found	Goat, Sheep	Turkey	[68]
*Theileria orientalis*	No	*Ixodes hexagonus*, *Ixodes ricinus*, *Rhipicephalus (B) annulatus*, *Rhipicephalus sanguineus* s.l.	Cattle, Dog	Buffalo, Cattle	Algeria, Egypt, Italy	[40,42,60,95]
*Theileria ovis*	No	*Ixodes ricinus*, *Rhipicephalus bursa*, *Rhipicephalus sanguineus* s.l.	Goat, Sheep	Buffalo, Goat, Sheep	Algeria, France, Greece, Palestine, Spain, Turkey	[22,37,44,50,65,66,67,68,81,97]
*Theileria uilenbergi*	No	data not found	data not found	Buffalo, Goat, Sheep	Egypt, Turkey	[68,89]
*Theileria sergenti*	No	*Ixodes hexagonus*, *Ixodes ricinus*, *Rhipicephalus (B) annulatus*, *Rhipicephalus sanguineus* s.l.	Cattle, Dog	Cattle, Horse	Italy, Spain	[42,47,60,86]
**Bacteria**						
*Anaplasma*						
*Anaplasma* spp.		*Argas persicus*, *Hyalomma excavatum*, *Hyalomma marginatum*, *Rhipicephalus* spp., *Rhipicephalus (B) annulatus*, *Rhipicephalus bursa*, *Rhipicephalus sanguineus* s.l.	Cattle, Chicken, Dogs, Dromedary, Goats, Horse, Sheep	Cat, Cattle, Dog, Donkey, Dromedary, Goat, Horse, Pig, Sheep	Algeria, France, Greece, Italy, Morocco, Spain, Tunisia, Turkey, Palestine	[24,26,68,98,99,100,101,102,103,104,105,106,107,108,109,110,111]
*Anaplasma bovis*	No	data not found	data not found	Cattle, Goat, Sheep	Italy, Tunisia	[30,100]
*Anaplasma centrale*	No	*Rhipicephalus bursa*, *Rhipicephalus sanguineus* s.l.	Cattle, Dog	Cattle, Goat	Italy, Morocco, Turkey	[24,30,98,100,112,113]
*Anaplasma marginale*	No	*Haemaphysalis punctata*, *Rhipicephalus bursa*, *Rhipicephalus sanguineus* s.l.	Cattle, Dog	Buffalo, Cat, Cattle, Dog, Donkey, Goat, Horse, Pig, Sheep	Algeria, Egypt, France, Italy, Morocco, Tunisia, Turkey	[30,32,33,34,35,39,43,44,98,100,103,105,112,113,114,115]
*Anaplasma ovis*	No	*Haemaphysalis punctata*, *Rhipicephalus bursa*, *Rhipicephalus sanguineus* s.l.	Cattle, Dog, Horse, Goat, Sheep	Cattle, Dog, Goat, Horse, Sheep	Algeria, France, Italy, Morocco, Tunisia, Turkey	[44,60,66,68,100,101,105,113,115,116,117,118,119,120,121]
*Anaplasma phagocytophilum*	Yes	*Haemaphysalis sulcata*, *Hyalomma marginatum*, *Ixodes hexagonus*, *Ixodes ricinus*, *Rhipicephalus bursa*, *Rhipicephalus sanguineus* s.l.	Cat, Dog, Goat, Horse, Sheep	Cat, Cattle, Dog, Donkey, Goat, Horse, Sheep	Algeria, Croatia, Egypt, France, Greece, Italy, Morocco, Spain, Tunisia, Turkey	[24,32,46,51,53,55,63,68,78,96,100,104,105,112,115,117,120,122,123,124,125,126,127,128,129,130,131,132,133,134,135,136,137,138,139,140,141]
*Anaplasma platys*	Yes	*Hyalomma* spp., *Ixodes hexagonus*, *Ixodes ricinus*, *Rhipicephalus (B) annulatus*, *Rhipicephalus sanguineus* s.l.	Cattle, Dog, Dromedary, Goat, Horse, Sheep	Buffalo, Cattle, Dog, Dromedary	Algeria, Croatia, Cyprus, Egypt, Greece, Italy, Morocco, Palestine, Spain, Tunisia, Turkey	[22,23,35,44,51,60,69,71,72,101,104,108,111,113,116,123,133,140,142,143,144,145,146,147,148]
*Bartonella*						
*Bartonella* spp.		*Rhipicephalus (B) annulatus*, *Rhipicephalus bursa*, *Rhipicephalus sanguineus* s.l.	Cattle, Dog, Goat	Cat, Dog	Italy, Spain	[85,119,139,149,150]
*Bartonella clarridgeiae*	Yes	data not found	data not found	Cat	Italy	[151]
*Bartonella henselae*	Yes	*Ixodes ricinus*	Cat, Dog	Cat, Dog	Algeria, Cyprus, Greece, Italy, Spain	[69,99,126,138,140,144,150,151,152,153,154,155,156]
*Bartonella vinsonii*	Yes	data not found	data not found	Cattle, Dog, Goat	Greece, Morocco	[157,158]
*Bartonella vinsonii berkhoffi*	Yes	*Rhipicephalus sanguineus* s.l.	Dog	Dog	Italy, Spain	[72,78,140]
*Borrelia*						
*Borrelia* spp.		*Argas persicus*, *Hyalomma aegyptium*, *Rhipicephalus (B) annulatus*	Cattle, chicken, Dog, Goat, Horses, Sheep	Cattle	Algeria, Turkey	[44,102,159]
*Borrelia afzelii*	Yes	*Ixodes hexagonus*, *Ixodes ricinus*	Dog	data not found	Italy, Spain	[51,60]
*Borrelia burgdoferi sensus lato*	Yes	*Hyalomma marginatum*, *Ixodes hexagonus*, *Ixodes ricinus*, *Rhipicephalus (B) annulatus*, *Rhipicephalus bursa*	Cattle, Dog	Dog, Horse	Croatia, Italy	[53,60,110,128,149]
*Borrelia garinii*	Yes	*Ixodes ricinus*	Dog	data not found	Spain	[51]
*Borrelia theileri*	No	data not found	data not found	Goat, Sheep	Algeria	[66]
*Borrelia valaisiana*	Yes	*Ixodes ricinus*	Dog	data not found	Spain	[51]
*Chlamydia*						
*Chlamydia abortus*	Yes	*Ixodes ricinus*, *Rhipicephalus (B) annulatus*, *Rhipicephalus bursa*, *Rhipicephalus sanguineus* s.l.	Cat, Cattle, Dog, Goat, Sheep	data not found	Italy	[154,160]
*Chlamydophila*						
*Chlamydophila psittaci*	Yes	*Ixodes ricinus*, *Rhipicephalus sanguineus* s.l.	Cat, Cattle, Dog, Goat, Sheep	data not found	Italy	[154,160]
*Coxiella*						
*Coxiella burnetii*	Yes	*Argas persicus*, *Dermacentor marginatus*, *Haemaphysalis punctata*, *Haemaphysalis sulcata*, *Hyalomma* spp., *Hyalomma dromedarii*, *Hyalomma marginatum*, *Ixodes ricinus*, *Rhipicephalus bursa*, *Rhipicephalus sanguineus* s.l.	Cattle, Chicken, Dog, Dromedary, Goat, Rabbit, Sheep	Buffalo, Cat, Cattle, Dromedary, Goat, Horse, Sheep	Algeria, Cyprus, Egypt, Greece, Italy, Montenegro, Slovenia, Spain, Tunisia	[32,66,74,78,119,139,153,161,162,163,164,165,166,167,168,169,170,171,172,173,174,175,176,177]
*Ehrlichia*						
*Ehrlichia* spp.		*Haemaphysalis parva*, *Hyalomma* spp., *Hyalomma excavatum*, *Hyalomma marginatum*, *Ixodes hexagonus*, *Ixodes ricinus*, *Rhipicephalus (B) annulatus*, *Rhipicephalus bursa*, *Rhipicephalus sanguineus* s.l.	Buffalo, Cattle, Dog, Sheep	Cattle, Dog, Goat, Sheep	Egypt, France, Italy, Palestine, Spain, Turkey	[26,60,68,85,99,110,111,112,149,170]
*Ehrlichia canis*	Yes	*Dermacentor marginatus*, *Haemaphysalis punctata*, *Haemaphysalis sulcata* *Hyalomma* spp., *Hyalomma excavatum*, *Ixodes hexagonus*, *Ixodes ricinus*, *Ixodes ventalloi*, *Rhipicephalus (B) annulatus*, *Rhipicephalus bursa*, *Rhipicephalus sanguineus* s.l.	Buffalo, Cat, Cattle, Dog, Goat, Sheep	Cat, Dog	Algeria, Croatia, Cyprus, Egypt, France, Greece, Italy, Palestine, Spain, Turkey	[32,53,55,60,72,73,74,78,103,106,107,111,119,123,126,127,129,138,139,143,144,146,153,170,178,179,180,181,182]
*Ehrlichia equi*	Yes	data not found	data not found	Horse	Italy	[32]
*Ehrlichia ewingii*	Yes	data not found	data not found	Dog	Cyprus	[143]
*Ehrlichia minancensis*	No	*Hyalomma marginatum*, *Rhipicephalus bursa*	Cattle, Goat	data not found	France	[183]
*Candidatus*						
*Candidatus* Ehrlichia urmitei	No	*Rhipicephalus (B) annulatus*	Cattle, Goat, Horse, Sheep	data not found	Algeria	[44]
*Noehrlichia*						
*Noehrlichia mikurensis*	Yes	*Ixodes ricinus*	Cattle	data not found	Spain	[137]
*Francisella*						
*Francisella* spp.	Yes	*Hyalomma marginatum*, *Rhipicephalus (B) annulatus*, *Rhipicephalus bursa*	Cattle	data not found	Italy	[149]
*Leptopsira*						
*Leptopsira* spp.	Yes	data not found	data not found	Buffalo, Cattle, Dromedary, Sheep	Egypt	[184]
*Mycoplasma*						
*Mycoplasma* spp.		data not found	data not found	Goat, Sheep	Morocco	[109]
*Mycoplasma haemocanis*	No	data not found	data not found	Dog	Greece, Turkey	[26,52,185,186]
*Mycoplasma haemofelis*	No	data not found	data not found	Cat	Cyprus	[178]
*Candidatus*						
*Candidatus* Mycoplasma haemonutum	No	data not found	data not found	Cat	Cyprus	[178]
*Candidatus* Mycoplasma haematoparvum	No	data not found	data not found	Dog	Greece, Turkey	[26,52,185,186]
*Candidatus* Mycoplasma turicensis	No	data not found	data not found	Cat	Cyprus	[178]
*Rickettsia*						
*Rickettsia* spp.		*Dermacentor marginatus*, *Haemaphysalis sulcata*, *Hyalomma* spp., *Hyalomma aegyptium*, *Hyalomma detritum*, *Hyalomma dromaderii*, *Hyalomma impeltatum*, *Hyalomma marginatum*, *Ixodes ricinus*, *Rhipicephalus* spp. *Rhipicephalus (B) annulatus*, *Rhipicephalus bursa*, *Rhipicephalus pusillus*, *Rhipicephalus sanguineus* s.l.	Cat, Cattle, Chicken, Dog, Donkey, Dromedary, Goat, Horse, Sheep	Cat, Dog, Horse	Algeria, Egypt, Israel, Italy, Palestine, Spain, Tunisia, Turkey	[85,99,102,138,139,149,159,170,187,188,189,190,191,192,193]
*Rickettsia aeschlimannii*	Yes	*Hyalomma dromedarii*, *Hyalomma impeltatum*, *Hyalomma marginatum*, *Hyalomma rufipes*, *Ixodes ricinus*	Cattle, Dromedary, Goat	Dromedary, Horse	Egypt, France, Israel, Spain, Tunisia	[170,175,193,194,195,196,197,198]
*Rickettsia africae*	Yes	*Amblyomma variegatum*, *Hyalomma* spp., *Hyalomma anatolicum*, *Hyalomma dromedarii*, *Hyalomma excavatum*, *Hyalomma impeltatum*, *Hyalomma marginatum*, *Hyalomma turanicum*	Cattle, Donkey, Dromedary, Goat, Sheep	Dromedary	Egypt, France, Israel, Italy, Lebanon, Palestine, Tunisia, Turkey	[189,190,194,197,199,200,201]
*Rickettsia conorii*	Yes	*Hyalomma marginatum*, *Rhipicephalus bursa*, *Rhipicephalus sanguineus* s.l.	Cattle, Dog, Donkey, Goat, Sheep	Cat, Dog	Greece, Italy, Spain, Tunisia	[106,119,138,140,141,173,179,182,189,191,202,203,204,205,206]
*Rickettsia conorii israelensis*	Yes	*Rhipicephalus sanguineus* s.l.	Dog, Goat	data not found	Italy	[119,207]
*Rickettsia felis*	Yes	*Rhipicephalus sanguineus* s.l.	Cat, Dog, Sheep	data not found	Italy, Spain	[208,209]
*Rickettsia helvetica*	Yes	*Hyalomma impeltatum*, *Ixodes ricinus*, *Ixodes ventalloi*	Cat, Cattle, Dromedary	Dromedary	Algeria, Italy, Tunisia	[73,154,201,210]
*Rickettsia hoogstraalii*	Unknown	*Haemaphysalis parva*, *Haemaphysalis sulcata*	Dog, Sheep	data not found	Greece, Italy	[22,119]
*Rickettsia massiliae*	Yes	*Haemaphysalis adleri*, *Haemaphysalis parva*, *Rhipicephalus (B) annulatus*, *Rhipicephalus bursa* *Rhipicephalus pusillus*, *Rhipicephalus sanguineus* s.l.	Cattle, Dog, Goat, Horse, Sheep	Cat, Dog, Dromedary	Algeria, Cyprus, France, Greece, Israel, Italy, Lebanon, Palestine, Spain, Tunisia	[22,44,51,58,137,153,173,190,191,192,193,195,201,205,207,209,210,211,212,213,214,215,216,217]
*Rickettsia monacensis*	Yes	*Hyalomma impeltatum*, *Ixodes ricinus*, *Rhipicephalus sanguineus* s.l.	Cat, Cattle, Dog, Dromedary, Goat	Dromedary	Algeria, France, Greece, Italy, Spain, Tunisia	[51,137,195,201,210,216,218,219]
*Rickettsia raoultii*	Yes	*Dermacentor reticulatus*, *Ixodes ricinus*, *Rhipicephalus sanguineus* s.l.	Cattle, Dog	data not found	Algeria, Spain, Turkey	[200,210,220]
*Rickettsia rickettsii*	Yes	data not found	data not found	Dog	Italy	[141]
*Rickettsia rhipicephali*	Unkown	*Rhipicephalus bursa*, *Rhipicephalus sanguineus* s.l.	Cattle, Dog, Goat	data not found	Greece	[173,205]
*Rickettsia sibirica mongolotimonae*	Yes	*Hyalomma excavatum*	Goat, Sheep	data not found	Cyprus	[212]
*Rickettsia slovaca*	Yes	*Dermacento marginatus*, *Haemaphysalis punctata*	Cattle, Dog, Donkey, Sheep, Pig	Cattle, Goat, Sheep	France, Italy, Spain, Turkey	[189,192,193,195,200,209,221,222]
*Rickettsia typhi*	Yes	data not found	data not found	Cat	Greece	[155]
*Candidatus*						
*Candidatus* Rickettsia goldwasserii	Unknown	*Haemaphysalis adleri*, *Haemaphysalis parva*, *Rhipicephalus sanguineus* s.l.	Dog, Goat, Horse, Sheep	data not found	Palestine	[190]
*Candidatus* Rickettsia barbariae	Unknown	*Hyalomma dromedarii*, *Rhipicephalus (B) annulatus*, *Rhipicephalus bursa*, *Rhipicephalus sanguineus* s.l.	Cattle, Dog, Goat, Horse, Sheep	data not found	Cyprus, France, Italy, Lebanon, Palestine	[119,190,195,212]
**Viruses**						
*Capripoxvirus*						
Lumpy skin disease virus	Yes	*Amblyomma* spp., *Amblyomma hebreaum*, *Hyalomma truncatum*, *Rhipicephalus (B) annulatus*, *Rhipicephalus appendiculatus*, *Rhipicephalus (B) microplus*	Cattle	data not found	Egypt	[223]
*Flavivirus*						
Tick-borne encephalitis	Yes	*Ixodes ricinus*	Goat	Goat	Greece, Italy	[224,225]
*Orthonairovirus*						
Crimean-Congo Hemorrhagic Fever	Yes	*Dermacentor marginatus*, *Haemaphysalis parva*, *Hyalomma anatolicum*, *Hyalomma dromedarii*, *Hyalomma excavatum*, *Hyalomma lusitanicum*, *Hyalomma marginatum*, *Ixodes ricinus*, *Rhipicephalus* spp., *Rhipicephalus bursa* s.l., *Rhipicephalus sanguineus* s.l.	Buffalo, Cattle, Dromedary, Dog, Goat, Sheep	Buffalo, Cattle, Goat, Sheep	Albania, Greece, Egypt, Spain, Turkey	[184,226,227,228,229,230,231,232,233,234,235]

**Table 2:** TBP diversity in domestic animals or engorged ticks collected on these animals from the Mediterranean Basin.

Categories of Domestic Animals	Livestock									Pets		
Animal names (number of countries with data available)	Cattle (13) (*Bos taurus*) 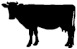	Goat (13) (*Capra hircus*) 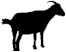	Sheep (15) (*Ovis aries*) 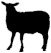	Dromedary (6) (*Camelus dromedarius*) 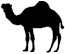	Horse (7) (*Equs caballus*) 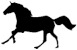	Buffalo (1) (*Bubalus* spp.) 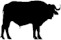	Chicken (2) (*Gallus gallus*) 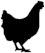	Donkey (1) (*Equs asinus*) 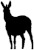	Pig (1) (*Sus domesticus*) 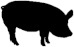	Dog (14) (*Canis lupus familiaris*) 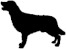	Cat (5) (*Felis silvestris*) 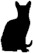	Rabbit (1) (*Oryctolagus* sp.) 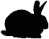
Pathogens found in ticks												
Number of studies	51	42	42	20	14	5	4	1	0	63	15	1
Number of TBPs found in ticks	Bacteria: 29 Parasite: 10	Bacteria: 25 Parasite: 5 Virus: 2	Bacteria: 21 Parasite: 7 Virus: 1	Bacteria: 8 Parasite: 2 Virus: 1	Bacteria: 12 Parasite: 1	Bacteria: 2 Virus: 1	Bacteria: 3	Bacteria: 4 Parasite: 4	0	Bacteria: 26 Parasite: 17 Virus: 1	Bacteria: 9 Parasite: 2	Bacteria: 1
Percentage of TBPs in ticks	59.1%	48.5%	43.9%	16.7%	19.7%	4.5%	4.5%	12.1%	0%	66.7%	16.7%	1.5%
**Pathogens found in animals**												
Number of studies	51	33	39	15	20	8	0	4	5	77	24	0
Number of TBPs found in animals	Bacteria: 13 Parasites: 13 Viruses: 2	Bacteria: 12 Parasites: 5 Viruses: 2	Bacteria: 11 Parasites: 10 Viruses: 1	Bacteria: 9 Parasites: 1	Bacteria: 9 Parasites: 6	Bacteria: 4 Parasites: 6 Viruses: 1	0	Bacteria: 3 Parasites: 2	Parasites: 2	Bacteria: 19 Parasites: 13	Bacteria: 15 Parasites: 5	0
Percentage of TBPs in animals	40.6%	27.5%	21.7%	14.5%	21.7%	15.9%	0%	7.2%	2.9%	46.4%	29%	0%

**Table 3:** Tick-borne pathogens from insular domestic animals or engorged ticks collected from these animals in the Mediterranean Basin.

Country	Island	Surface Area	Western Basin/Eastern Basin	Pathogen (Found in ^A^ for Positive Animal and/or ^T^ for Positive Tick)	Positive Ticks	Positive Tick Hosts	Positive Pathogen Hosts	References
Cyprus	Cyprus	9251 km^2^	Eastern	*A. platys*^A^, *B. vogeli*^A^, *Ba. henselae*^A^, *C. burnetii*^T^, *E. canis*^A^, *E. ewingii*^A^, *H. canis*, *H. felis*^A^*M. haemofelis*^A^, *Candidatus* M. haemotumum ^A^, *Candidatus* M. turicensis ^A^, *R. aeschlimanii* ^T^, *R. hoostraalii* ^T^, *R. sibirica mongolotimonae* ^T^ and *Candidatus* R. barbariae ^T^	*H. punctata*, *H sulcata*, *Hyalomma* spp., *Hy. excavatum*, *Hy. marginatum*, *Hy. rufipes*, *I. gibbosus*, *Rh. bursa* and *Rh. sanguineus* s.l.	Cattle, Goat and Sheep	Cat and Dog	[69,143,177,178,212]
France	Corsica	8722 km^2^	Western	*Anaplasma* spp. ^A,T^, *A. ovis* ^A^, *A. marginale* ^A,T^, *A. phagocytophilum* ^T^, *B. bigemina* ^T^, *B. canis* ^A,T^, *B. vogeli* ^A,T^, *Ba. henselae* ^T^, *Bo. afzelii* ^T^, *Bo. burgdoferi* s.l. ^T^, *Bo. miyamotoi* ^T^, *E. canis* ^T^, *E. minancensis*, *R. aeschlimanii* ^T^, *R. africae* ^T^, *R. helvetica* ^T^, *R. massiliae* ^T^, *R. monacensis* ^T^, *R. slovaca* ^T^, *Candidatus* R. barbariae ^T^, *T. annae* ^A^ and *T. equi ^T^*	*A. variegatum*, *D. marginatus*, *D. reticulatus*, *I. ricinus*, *Hy. aegyptium*, *Hy. marginatum*, *Hy. rufipes*, *Rh. bursa* and *Rh. sanguineus* s.l.	Cat, Cattle, Dog and Sheep	Cattle, dog, Horse, goat and sheep	[56,103,183,195,198,199,272]
Greece	Crete	8450 km^2^	Eastern	*Ba. henselae*^A^, *Ba. vinsonii*^T^, *R. felis*^A^*and R. Typhi*^A^	*Rh. bursa* and *Rh. sanguineus* s.l.	Goat, Cattle	Cat	[155,157]
	Ios	109 km^2^	Eastern	*Anaplasma* spp. ^A^, *B. canis* ^A^, *Bo. burgdoferi* ^A^, *E. canis* ^A^ *and R. conorii* ^A^	data not found	data not found	Dog	[106]
	Mykonos	85.5 km^2^	Eastern	*Ba. henselae ^A^ and Rickettsia* spp. ^A^	data not found	data not found	Cat	[155]
	Santorini	76.19 km^2^	Eastern	*Anaplasma* spp. ^A^, *B. canis* ^A^, *Bo. burgdoferi* ^A^, *E. canis* ^A^ *and R. conorii* ^A^	data not found	data not found	Dog	[106]
	Skiathos	49.9 km^2^	Eastern	*Anaplasma* spp.^A^, *B. canis* ^A^, *Bo. burgdoferi* ^A^, *E. canis* ^A^ *and R. conorii* ^A^	data not found	data not found	Dog	[106]
	Skopelos	96.3 km^2^	Eastern	*Ba. henselae*^A^*and Rickettsia* spp. ^A^	data not found	data not found	Cat	[155]
	Tinos	197 km^2^	Eastern	*Anaplasma* spp. ^A^, *B. canis* ^A^, *Bo. burgdoferi* ^A^, *E. canis* ^A^ *and R. conorii* ^A^	data not found	data not found	Dog	[106]
Italy	Aeolian Island	114.7 km^2^	Western	*Anaplasma* spp. ^A^, *B. vogeli* ^A^, *Bartonella* spp. ^A^, *Ba. clarridgeiae* ^A^, *Ba. henselae* ^A^, *Ehrlichia canis* ^A^, *H. canis* ^A^, *H. felis* ^A^, *R. helvetica* ^A^ *and R. monacensis* ^A^	*I. ricinus*, *I. ventalloi. Rh. pusillus* and *Rh. sanguineus* s.l.	Cat	Cat and Dog	[73,150]
	Sardinia	24090 km^2^	Western	*A. phagocytophilum*^A^, *A. ovis*^A^, *B. bigemina*^T^*Bartonella* spp. ^T^, *Ba. henselae* ^A,T^, *Chlamydia abortus* ^T^, *Chlamydophila psittaci* ^T^, *C. burnetii* ^A,T^, *E. canis* ^T^, *Rickettsia* spp. ^T^, *R. aeschlimannii* ^T^, *R. conorii israelensis* ^T^, *R. helvetica* ^T^, *R. hoogstralii ^T^*, *R. massiliae* ^T^, *R. slovaca* ^T^, *Candidatus R. barbariae* ^T^, *T. buffeli* ^T^, *T. equi* ^T^, *T. orientalis* ^T^and *T. sergenti* ^T^	*D. marginatus*, *H. punctata*, *H. sulcata*, *Hy. marginatum*, *I. festai. Rhipicephalus* spp., *Rh (B). annulatus*, *Rh. bursa*, *Rh.* sanguineus *s.l.*	Cat, Cattle, Dog, Goat, Horse, Pig and Sheep	Cat, Dog, Goat, Horse and Sheep	[38,119,122,139,156,160,171,207,215,221]
	Sicily	25711 km^2^	Western	*Anaplasma* spp. ^A^, *A. marginale* ^A^, *A. phagocytophilum* ^A^, *A. platys* ^A^, *A. ovis* ^A^, *Babesia* spp. ^A^, *B. bigemina* ^A^, *B. bovis* ^A^, *B. caballi* ^A^, *B. canis* ^A^, *B. microti* ^A^, *B. vogeli* ^T^, *Ba. claridgeiae* ^A,T^, *Ba. henselae* ^A^, *Bo. burgdoferi* s.l ^A^, *Cercopithifilaria* spp. ^A,T^, *C. burnetii* ^A^, *Ehrlichia* spp. ^A^, *E. canis* ^A^, *E. equi* ^A^, *Hepatozoon* spp. ^A,T^, *Rickettsia* spp. ^A,T^, *R. aeschlimannii* ^T^, *R. africae* ^T^, *R. conorii* ^A,T^, *R. felis* ^A^, *R. helvetica* ^T^, *R. monacensis* ^T^, *R. rickettsii* ^A^, *R. slovaca* ^T^, *Theileria* spp. ^A^, *T. annulata* ^A^ *and T. equi* ^A^	*D. reticulatus*, *D. marginatus*, *H. punctata*, *Hy. lusitacum*, *Hy. marginatum*, *Ixodes* spp., *I. ricinus*, *I. ventalloi*, *Rh. bursa*, *Rh. pusillus*, *Rh. sanguineus* s.l.	Cat, Cattle, Dog, Donkey and Sheep	Cat, Cattle, Dog, Donkey, Goat, Horse, Pig and Sheep	[32,91,105,115,131,138,145,146,151,179,189,273,274]
Spain	Mallorca	3640 km^2^	Western	*A. phagocytophilum*^A^, *Ba. henselae*^A^, *Ba. vinsonii berkhoffi*^A^, *E. canis*^A^*and R. conorii*^A^	data not found	data not found	Cat and Dog	[140,182]

## Data Availability

The authors confirm that the data supporting the findings of this study are available within the article and its supplementary material. Raw data that support the findings of this study are available from the corresponding author, upon reasonable request.

## Supplementary Materials

The following supporting information can be downloaded at: https://www.mdpi.com/article/10.3390/microorganisms10061236/s1, Table S1: Positive engorged ticks for TBPs collected on domestic animals and their distribution in the Mediterranean Basin; Table S2: TBP species reported in domestic animals or engorged ticks collected on these animals from the Mediterranean Basin.
