# Supplementary material for: Distribution of Tick-Borne Pathogens in Domestic Animals and Their Ticks in the Countries of the Mediterranean Basin between 2000 and 2021: A Systematic Review
Source: Microorganisms. 2022 Jun 16;10(6):1236. doi: 10.3390/microorganisms10061236 (PMC9228937; doi:10.3390/microorganisms10061236)
Supplement: Supplementary file 1 [file microorganisms-10-01236-s001.zip › microorganisms-1737112-supplementary.pdf]

microorganisms

MDPI

Supplementary materials

Table S1. Positive engorged ticks for TBPs collected on domestic animals and their distribution in the Mediterranean Basin.

Tick genus	Number of tick species and tick species		Stage	Number of countries with data and countries		Number of tick host species with data and host species		Number of pathogenic genera/species and pathogens detected in ticks ([b]bacteria, [p]parasite, [v]virus)	
Rhipicephalus	7	***Rh (Bo). annulatus, Rh. appendiculatus, Rh. bursa, Rh (Bo). microplus, Rh. pusillus, Rh. sanguineus* s.l.**	**Larva, nymph and adult**	13	Algeria, Cyprus, Egypt, France, Greece, Italy, Lebanon, Morocco, Palestine, Spain, Tunisia, Turkey	8	Buffalo, Cat, Cattle, Dog, Donkey, Goat, Horse and Sheep	13/57	*Anaplasma* spp.[b], *A. centrale*[b], *A. marginale*[b], *A. ovis*[b], *A. phagocytophilum*[b], *A. platys*[b], *Babesia* spp.[p], *B. bigemina*[p], *B. bovis*[p], *B. canis*[p], *B. capreoli*[p], *B. divergens*[p], *B. gibsoni*[p], *B. microti*[p], *B. ovis*[p], *B. venatorum*[p], *B. vogeli*[p], *Bartonella* spp[b]., *Ba. caballi*[b], *Ba. vinsonii*[b], *Ba. vinsonii berkhoffi*[b], *Borrelia* spp.[b], *Bo. burgdoferi* s.l.[b], *Cercopithifilaria bainae*[p], *Chlamydia abortus*[b], *Chlamydophila psittaci*[b], *Coxiella burnetii*[b], Crimean-Congo hemorrhagic fever virus[v], *Ehrlichia* spp.[b], *E. canis*[b], *E. minacensis*[b], *Candidatus* E. urmitei[b], *Francisella* spp.[b], *Hepatozoon spp*[p], *Hepatozoon canis*[p], *Hepatozoon felis*, lumpy skin disease virus[v], *Rickettsia* spp.[b], *R. conorii*[b], *R. conorii israelensis*[b], *R. felis*[b], *R. massiliae*[b], *R. monacensis*[b], *R. raoultii*[b], *R. rhipicephali*[b], *Candidatus Rickettsia* goldwasserii[b], *Candidatus* R. barbariae, *Theileria* spp.[p], *Theileria annae*[p], *T. annulata*[p], *T. buffeli*[p], *T. cervi*[p], *T. equi*[p], *T. orientalis*[p], *T. ovis*[p], *T. sergenti*[p]
Ixodes	3	*I. hexagonus, I. ricinus* and *I. ventalloi*	Larva, nymph and adult	9	Algeria, France, Greece, Italy, Morocco, Slovenia, Spain, Turkey	6	Cat, Cattle, Dog, Goat, Horse and Sheep	12/36	*Anaplasma phagocytophilum*[b], *A. platys*[b], *Babesia bovis*[p], *B. canis*[p], *B. capreoli*[p], *B. divergens*[p], *B. gibsoni*[p], *B. microti*[p], *B. ovis*[p], *B. venatorum*[p], *B. vogeli*[p], *Bartonella henselae*[b], *Borrelia* spp.[b], *Bo. afzelii*[b], *Bo. burgdoferi* s.l.[b], *Bo. garinii*[b], *Bo. valaisiana*[b], *Chlamydia abortus*[b], *Chlamydophila psittaci*[b], *Coxiella burnetii*[b], Crimean-Congo hemorrhagic fever virus[v], *Ehrlichia* spp.[b], *E. canis*[b], *Noehrlichia mikurensis*[b], *Hepatozoon canis*[p], *Rickettsia* spp.[b], *R. helvetica*[b], *R. monacensis*[b], *R. raoultii*[b], *Theileria annae*[p], *T. buffeli*[p], *T. cervi*[p], *T. equi*[p], *T. orientalis*[p], *T. ovis*[p], *T. sergenti*[p], Tick-borne encephalitis virus[v]
Hyalomma	11	*Hy. aegyptium, Hy. anatolicum, Hy. deritum, Hy. dromedarii, Hy. excavatum, Hy. impeltatum, Hy.*	Larva, nymph and adult	12	Algeria, Cyprus, Egypt, France, Greece, Israel, Italy, Lebanon, Palestine, Spain, Tunisia, Turkey	9	Buffalo, Cat, Cattle, Dog, Donkey Dromedary, Goat, Horse and Sheep	11/25	*Anaplasma* spp.[b], *A. phagocytophilum*[b], *A. platys*[b], *Babesia* spp.[p], *B. bigemina*[p], *B. bovis*[p], *Borrelia* spp.[b], *Bo. burgdoferi* s.l.[b], *Coxiella burnetii*[b], Crimean-Congo hemorrhagic fever virus[v], *Ehrlichia* spp.[b], *E. canis*[b], *E. minacensis*[b], *Francisella* spp.[b], lumpy skin disease virus[v], *Rickettsia* spp.[b], *R. aeschlimannii*[b], *R. africae*[b], *R. helvetica*[b], *R. monacensis*[b], *R. sibirica mongolotimonae*[b], *Theileria* spp.[p], *Theileria annulata*[p], *T. buffeli*[p], *T. equi*[p]

		lusitanicum, *Hy. marginatum*, *Hy. rufipes*, *Hy. truncatum* and *Hy. turanicus*							
Haemaphysalis	5	*H. adleris*, *H. concinna*, *H. parva*, *H. punctata* and *H. sulcata*	Larva, nymph and adult	7	Algeria, Greece, Italy, Palestine, Slovenia, Spain, Turkey	8	Cat, Cattle, Dog, Donkey, Horse, Goat, Pig and Sheep	8/16	*Anaplasma marginale* [b], *A. ovis* [b], *A. phagocytophilum* [b], *B. ovis* [p], *Coxiella burnetii* [b], Crimean-Congo hemorrhagic fever virus [v], *Ehrlichia* spp. [b], *E. canis* [b], *Hepatozoon canis* [p], *H. felis* [p], *Rickettsia* spp. [b], *R. hoogstraalii* [b], *R. massiliae* [b], *R. slovaca* [b], *Candidatus* Rickettsia goldwasserii [ib], *Theileria buffeli* [p]
Dermacentor	2	*D. marginatus* and *D. reticulatus*	Larva, nymph and adult	8	Algeria, France, Greece, Italy, Slovenia, Spain, Turkey	6	Cattle, Dog, Donkey, Goat, Pig and Sheep	6/12	*Babesia bovis* [p], *B. canis* [p], *B. gibsoni* [p], *B. microti* [p], *B. ovis* [p], *B. vogeli* [p], Crimean-Congo hemorrhagic fever virus, *Coxiella burnetii* [b], *Rickettsia spp* [b], *R. raoultii* [b], *R. slovaca* [b], *Theileria annae* [p]
Argas	1	*Ar. persicus*	Adult	2	Algeria, Turkey	2	Chicken and Rabbit	3/3	*Anaplasma* spp.[b], *Borrelia* spp. [b], *Coxiella burnetii* [b]
Amblyomma	2	*A. hebreaum* and *A. variegatum*	Adult	3	Egypt, France, Israel	1	Cattle	2/2	Lumpy skin disease virus[v], *R. africae* [b]

Table S2. TBP species reported in domestic animals or engorged ticks collected on these animals from the Mediterranean Basin.

Categories of domestic animals	Animal names	Pathogens found in ticks ([b]bacteria, [p]parasite, [v]virus)	Pathogens found in animals ([b]bacteria, [p]parasite, [v]virus)
Livestock	Cattle	*Anaplasma* spp.[b], *A. centrale* [b], *A. marginale* [b], *A. ovis* [b], *A. platys* [b], *Babesia* spp. [p], *B. bigemina* [p], *B. bovis* [p], *B. caballi* [p], *Bartonella* spp [b]., *Borrelia* spp [b]. *Bo. burgdoferi* s.l. [b], *Chlamydia. abortus* [b], *Chlamydophila psittaci* [b], *Coxiella burnetii* [b], *Ehrlichia* spp.[b], *E. canis* [b], *E. minancensis* [b], *Candidatus.* E. *urmitei* [b], *Francisella* spp. [b], *Neoehrlichia mikurensis* [b], *Rickettsia* spp.[b], *R. aeschlimannii* [b], *R. africae* [b], *R. conorii* [b], *R. helvetica* [b], *R. massiliae* [b], *R. monacensis* [b], *R. raoultii* [b]. *R. rhipicephali* [b]. *R. slovaca* [b], *Candidatus R. barbariae* [b], *Theileria* spp. [p], *T. annulata* [p], *T. buffeli* [p], *T. equi* [p], *T. orientalis* [p], *T. sergenti* [p]	*Anaplasma* spp. [b], *A. bovis* [b], *A. centrale* [b], *A. marginale* [b], *A. ovis* [b], *A. phagocytophilum* [b], *A. platys* [b], *Babesia bigemina* [p], *B. bovis* [p]. *B. divergens* [p], *B. major* [p], *B. occultans* [p], *B. ovis* [p], *Bartonella vinsonii* [b], *Borrelia* spp. [b]¸ Crimean-Congo hemorrhagic fever virus [v], *Coxiella burnetii* [b], *Ehrlichia* spp. [b], *Leptospira* spp. [b], Lumpy skin disease virus [v], *Rickettsia slovaca* [b], *Theileria* spp. [p], *T. annae* [p], *T. annulata* [p], *T. buffeli* [p], *T. equi* [p], *T. orientalis* [p], *T. sergenti* [p]
	Goat	*Anaplasma* spp.[b], *A. ovis* [b], *A. phagocytophilum* [b], *A. platys* [b], *Babesia bovis* [p], *B. microti* [p], *B. ovis* [p], *B. vogeli* [p], *Bartonella* spp. [b], *Borrelia* spp. [b], *Chlamydia abortus* [b], *Chlamydophila psittaci* [b], Crimean-Congo hemorrhagic fever virus [v], *Coxiella burnetii* [b], *Ehrlichia canis* [b], *E. minancensis* [b], *Candidatus* E. urmitei [b], *Rickettsia* spp. [b], *R. aeschlimannii* [b], *R. africae* [b], *R. conorii* [b], *R. conorii israelensis* [b], *R. massiliae* [b], *R. monacensis* [b], *R. rhipicephali* [b], *R. sibirica mongolotimonae* [b], *Candidatus* R. goldwassenii [b], *Candidatus* R. barbariae [b], *Theileria buffeli* [p], *T. ovis* [p], Tick-borne encephalitis virus [v]	*Anaplasma* spp. [b], *A. bovis* [b], *A. centrale* [b], *A. marginale* [b], *A. ovis* [b], *A. phagocytophilum* [b], *Babesia* spp., *Bartonella vinsonii* [b], *Borrelia theileri* [b], Crimean-Congo hemorrhagic fever virus [v], *Coxiella burnetii* [b], *Ehrlichia* spp. [b], *Mycoplasma* spp. [b], *Rickettsia slovaca* [b], *Theileria* spp. [p], *T. annulata* [p], *T. luwenshuni* [p], *T. ovis* [p], *T. uilenberg* [p], Tick-borne encephalitis virus [v]
	Sheep	*Anaplasma* spp. [b], *A. ovis* [b], *A. phagocytophilum* [b], *A. platys* [b], *Babesia bigemina* [p], *B. bovis* [p], *B. microti* [p], *B. ovis* [p], *B. vogeli* [p], *Borrelia* spp. [b], *Chlamydia abortus* [b], *Chlamydophila psittaci* [b],	*Anaplasma* spp. [b], *A. bovis* [b], *A. marginale* [b], *A. ovis* [b], *A. phagocytophilum* [b], *Babesia* spp. [p], *B. bigemina* [p], *B. motasi* [p], *B. ovis* [p], *Borrelia theileri* [b], Crimean-Congo hemorrhagic

		Crimean-Congo hemorrhagic fever virus [v], *Coxiella burnetii* [b], *Ehrlichia* spp. [b], *E. canis* [b], *Candidatus* E. urmitei [b], *Rickettsia* spp. [b], *R. africae* [b], *R. conorii* [b], *R. felis* [b], *R. hoogstraalii* [b], *R. massiliae* [b], *R. sibirica mongolotimonae* [b], *R. slovaca* [b], *Candidatus* R. goldwassenii [b], *Candidatus* R. barbariae [b], *Theileria annulata* [p], *T. ovis* [p]	fever virus [v], *Coxiella burnetii* [b], *Ehrlichia* spp. [b], *Leptospira* spp. [b], *Mycoplasma* spp. [b], *Rickettsia slovaca* [b], *Theileria* spp. [p], *T. annulata* [p], *T. lestoquardi* [p], *T. luwenshuni* [p], *T. ovis* [p], *T. uilenbergi* [p]
	Dromedary	*Anaplasma* spp. [b], *A. platys* [b], Crimean-Congo hemorrhagic fever virus [v], *Coxiella burnetii* [b], *Rickettsia* spp. [b], *R. aeschlimannii* [b], *R. africae* [b], *R. helvetica* [b], *R. monacensis* [b], *Theileria* spp. [p], *T. annulata* [p]	*Anaplasma* spp. [b], *A. platys* [b], *Coxiella burnetii* [b], *Leptospira* spp. [b], *Rickettsia aeschlimannii* [b], *R. massiliae* [b], *R. africae* [b], *R. helvetica* [b], *R. monacensis* [b], *Theileria annulata* [p]
	Horse	*Anaplasma* spp. [b], *A. ovis* [b], *A. phagocytophilum* [b], *A. platys* [b], *Borrelia* spp. [b], *Candidatus* Ehrlichia urmitei [b], *Rickettsia* spp. [b], *R. massiliae* [b], *Candidatus* R. goldwassenii [b], *Candidatus* R. barbariae [b], *Theileri equi* [p]	*Anaplasma* spp. [b], *A. marginale* [b], *A. ovis* [b], *A. phagocytophilum* [b], *Babesia caballi* [p], *B. equi, Borrelia burgdoferi* s.l. [b], *Coxiella burnetii* [b], *Ehrlichia equi* [b], *Rickettsia* spp. [b], *R. aechlimannii* [b], *Theileria annae* [p], *T. buffeli* [p], *T. equi* [p], *T. sergenti* [p]
	Buffalo	Crimean-Congo hemorrhagic fever virus [v], *Ehrlichia* spp. [b], *E. canis* [b]	*Anaplasma marginale* [b], *A. platys* [b], *Babesia bovis* [p], Crimean-Congo hemorrhagic fever virus [v], *Coxiella burnetii* [b], *Leptospira* spp. [b], *Theileria annulata* [p], *T. lestoquardi* [p], *T. orientalis* [p], *T. ovis, T. uilenbergi* [p]
	Chicken	*Anaplasma* spp. [b], *Borrelia* spp. [b], *Rickettsia* spp. [b]	-
	Donkey	*Rickettsia* spp. [b], *R. africae* [b], *R. conorii* [b], *R. slovaca* [b], *Theileria* spp. [p], *T. annae* [p], *T. annulata* [p], *T. equi* [p]	*Anaplasma* spp. [b], *A. marginale* [b], *A. phagocytophilum* [b], *Babesia* spp. [p], *B. caballi* [p]
	Pig		*Babesia* spp. [p], *Theileria* spp. [p]
Pet	Dog	*Anaplasma* spp. [b], *A. centrale* [b], *A. marginale* [b], *A. ovis* [b], *A. phagocytophilum* [b], *A. platys* [b], *Babesia bigemina* [p], *B. canis* [p], *B. capreoli* [p], *B. divergens* [p], *B. gibsoni* [p], *B. microti* [p], *B. ovis* [p], *B. venatorum* [p], *B. vogeli* [p], *Bartonella* spp. [b], *Ba. henselae* [b], *Ba. vinsonii berkhoffi* [b], *Borrelia* spp. [b], *Bo. afzelii* [b], *Bo. bugdoferi* s.l. [b],	*Anaplasma* spp. [b], *A. marginale* [b], *A. ovis* [b], *A. phagocytophilum* [b], *A. platys* [b], *Babesia* spp. [p], *B. caballi* [p], *B. canis* [p], *B. gibsoni* [p], *B. microti* [p], *B. vogeli* [p], *Bartonella* spp. [b], *Ba. henselae* [b], *Ba. vinsonii* [b], *Ba. vinsonii berkhoffi* [b], *Borrelia bugdoferi* s.l. [b], *Cercopithifilaria bainae* [p], *Ehrlichia*

		Bo. garinii [b], *Bo. valaisiana* [b], *Cercopithifilaria bainae* [p], Crimean-Congo hemorrhagic fever virus [v], *Chlamydia abortus* [b], *Chlamydophila psittaci* [b], *Coxiella burnetii* [b], *Ehrlichia* spp. [b], *E. canis* [b], *Hepatozoon* spp. [p], *H. canis* [p], *H. felis* [p], *Rickettsia* spp. [b], *R. conorii* [b], *R. conorii israelensis* [b], *R. felis* [b], *R. hoogstraalii* [b], *R. massiliae* [b], *R. monacensis* [b], *R. raoultii* [b], *R. rhipicephali* [b], *R. slovaca* [b], *Candidatus* R. goldwassenii [b], *Candidatus* R. barbariae [b], *Theileria annae* [p], *T. buffeli* [p], *T. equi* [p], *T. orientalis* [p], *T. sergenti* [p]	spp. [b], *E. canis* [b], *E. ewingii* [b], *Hepatozoon* spp. [p], *H. canis* [p], *Mycoplasma haemocanis* [b], *Candidatus* M. haematoparvum [b], *Rickettsia* spp. [b], *R. conorii* [b], *R. massiliae* [b], *R. rickettsia* [b], *Theileria* spp. [p], *T. annae* [p]. *T. cervi* [p], *T. equi* [p]
	Cat	*Anaplasma phagocytophilum* [b], *Babesia vogeli* [p], *Bartonella henselae* [b], *Chlamydia abortus* [b], *Chlamydophila psittaci* [b], *Ehrlichia canis* [b], *Rickettsia* spp. [b], *R. felis* [b], *R. helvetica* [b], *R. monacensis* [b], *Theileria buffeli* [p]	*Anaplasma* spp. [b], *A. marginale* [b], *A. phagocytophilum* [b], *Babesia microti* [p], *Bartonella* spp. [b], *Ba. clarridgeiae* [b], *Ba. henselae* [b], *Coxiella burnettii* [b], *Ehrlichia canis* [b], *Hepatozoon* spp. [p], *H. canis* [p], *H. felis* [p], *Mycoplasma haemofelis* [b], *Candidatus* M. haemonotum [b], *Candidatus* M. turicensis [b], *Rickettsia* spp. [b], *R. conorii* [b], *R. massiliae* [b], *R. typhi* [b], *Theileria* spp. [p]
	Rabbit	*Coxiella burnetii* [b]	
